# A Stop Smoking In Schools Trial in three culturally different middle-income countries (ASSIST global): protocol for a randomised feasibility study

**DOI:** 10.1136/bmjopen-2024-096963

**Published:** 2025-06-22

**Authors:** Yin Nwe Soe, Meigan Thomson, Kate Reid, Laurence Moore, Bagas Suryo Bintoro, Bin Dong, Sally Good, Peijin Hu, Emily Long, Nino Jose Mateo, Nicola McMeekin, Retna Siwi Padmawati, Yayi Suryo Prabandari, Anthony Purvis, Maria Guadalupe Salanga, Sean Semple, Charisse Tan Llorin, Jing-Yi Wang, Sharon Anne Simpson

**Affiliations:** 1 MRC/CSO Social and Public Health Sciences Unit, School of Health and Wellbeing, University of Glasgow, Glasgow, UK; 2 School of Education, University of Glasgow, Glasgow, UK; 3 Faculty of Medicine, Public Health and Nursing, Universitas Gadjah Mada, Yogyakarta, Indonesia; 4 Institute of Child and Adolescent Health, School of Public Health, Peking University Health Science Center, Beijing, China; 5 Evidence-to-Impact, Bristol, UK; 6 Counseling and Educational Psychology Department, Br. Andrew Gonzalez FSC College of Education, De La Salle University, Manila, Philippines; 7 Health Economics and Health Technology Assessment, University of Glasgow Institute of Health and Wellbeing, Glasgow, UK; 8 Department of Psychology, College of Liberal Arts, De La Salle University, Manila, Philippines; 9 Institute for Social Marketing, University of Stirling, Stirling, UK; 10 Social Development Research Center, De La Salle University, Manila, Philippines

**Keywords:** Smoking Reduction, Randomized Controlled Trial, Feasibility Studies, Schools, Adolescents

## Abstract

**Introduction:**

Around 80% of the world’s smokers live in lower-middle income countries and smoking rates in China, Philippines and Indonesia are very high. Evidence suggests that most people begin smoking or become habitual smokers before reaching adulthood. This highlights the need for a smoking prevention intervention focused on young people. ASSIST (A Stop Smoking In Schools Trial) is a ‘peer-led’, school-based smoking prevention intervention, shown to be effective in the UK. The aim of the study is to assess the feasibility of conducting a full-scale effectiveness evaluation of an adapted version of the ASSIST intervention in China, Indonesia or the Philippines. However, due to issues with obtaining relevant approvals, China was removed from the trial with the approval of the funder and Trial Steering Committee, and the study will only be completed in Indonesia and the Philippines.

**Methods and analysis:**

A feasibility mixed-methods cluster randomised controlled trial in 10 schools (six intervention, four control) in each of the two countries. Participants will be students aged c13–14 in mainstream (‘lower secondary’) schools. In addition to their usual education on smoking, intervention schools will receive the ASSIST intervention which is based on ‘diffusion of innovation’ theory, with new norms and behaviours promoted through: (1) peer modelling by locally influential individuals; and (2) information disseminated by them through their social networks. Control schools will continue with their usual education around smoking prevention.

The key outcome of the study is whether prespecified progression criteria relating to recruitment, retention, acceptability and feasibility have been met in order to progress to a larger cluster randomised controlled effectiveness trial in one or more of the countries. A mixed-methods process evaluation will assess acceptability, feasibility and fidelity of intervention delivery, exposure to and reach of the intervention. The feasibility of trial processes including outcome measurement will be assessed. An economic evaluation will estimate the costs of the ASSIST intervention. Statistical analyses will focus on feasibility criteria, and qualitative data will be analysed using a framework approach. Outcomes assessed will include self-reported smoking behaviour (own and that of friends and family); vaping and other forms of nicotine use; smoking-related attitudes and knowledge; smoking norms; self-esteem; self-efficacy; (all at baseline and 7 month follow-up) and exhaled carbon monoxide concentration (at follow-up only).

**Ethics and dissemination:**

The trial has been approved by the University of Glasgow College of Medical, Veterinary and Life Sciences (MVLS) Ethics Committee (ref: 200210204), the De La Salle University Research Ethics Review Committee (ref: 2023-012C) and the Medical and Health Research Ethics Committee (MHREC); Faculty of Medicine, Public Health and Nursing; Universitas Gadjah Mada (ref: KE/FK/1205/EC/2022). The trial is sponsored by the University of Glasgow (Head of Research Regulation and Compliance—debra.stuart@glasgow.ac.uk). The sponsor will not have input in data collection, management, analysis and interpretation; write up and submissions for publication.

The study findings will be disseminated through peer-reviewed publications in expert journals and conference presentations and targeted communications to schools, policymakers and the public.

**Trial registration number:**

ISRCTN99140476.

**STRENGTHS AND LIMITATIONS OF THIS STUDY**

- The study includes a mixed-methods process evaluation which will provide rich triangulated data to inform questions of feasibility and acceptability as well as required contextual adaptations.
- The intervention being tested is theory based, and we will update and refine the programme theory as part of this study.
- A limitation of the study is that blinding of participants and intervention delivery staff will not be possible.
- This feasibility trial is not designed to identify an estimate of effect and thus a standard power calculation is not appropriate.

## Introduction

 Tobacco is the world’s leading cause of avoidable poor health and premature death.^1^ In higher-income countries, tobacco use peaked in the 1960s/1970s, falling as tobacco control programmes were introduced in recognition of its health harms.^2^ Around 80% of the world’s smokers live in low-income and middle-income countries (LMICs). ^3 4^This study is set in Indonesia (population 277 million) and the Philippines (population 117 million), both are LMICs in South East Asia, but they have very different cultural and contextual influences. In the Philippines, Christianity is the main religion (89%) and in Indonesia, 87% of the population are Muslims. In the Philippines, school is compulsory for 13 years from age 5 and in Indonesia, it is compulsory to attend 12 years of schooling. According to the World Bank, in 2024, the poverty rate is 9% in Indonesia and 15.5% in 2023 in the Philippines.^1^

The prevalence of smoking in Indonesia and the Philippines is high, with the latest available data on current tobacco use in those aged 15 years and older (male/female) reported in 2021 as: Indonesia 60.4%/1.8%; Philippines 41.6%/6.5%.^1^ The most recent surveys from 13 to 15 year olds again highlight high male rates in the two countries: rates of any tobacco use in males/females of 35.6%/3.5% in Indonesia^5^ and rates of any cigarette smoking of 18.3%/6.9% in the Philippines.^6^ Prevalence rates vary in LMICs for under 15 s, it is estimated on average that 13.6% of 12–15 year olds are current smokers (defined as smoking on at least 1 out of the past 30 days).^7^

High adult smoking rates and strong smoking cultures mean large numbers of family members smoke, and although health risks may be acknowledged, smoking is often viewed as the norm, including as a masculine rite of passage.^8^,^10^ Cigarette advertising is permitted in some settings in Indonesia,^11^ while the Philippines have adopted stronger policies in the past decade.^12^,^14^ There are few or no smoking cessation programmes for adolescents in these countries.^15^ However, while tobacco control policies are crucial, public health practitioners recognise the need for interventions at *multiple* socioecological levels.^16^

The very high smoking rates in the two countries highlight the need for *youth* prevention, given evidence that few people begin smoking or become habitual smokers in adulthood.^17^ While both countries have smoke-free school rules, there are variations in enforcement and in the provision of school-based smoking prevention programmes. When implemented, these programmes are often infrequent, inconsistent and adult-led, which are less effective than those led by peers.^15^ Most people begin smoking before the age of 18.^18^ Research shows those who begin smoking during adolescence are more likely to become habitual smokers in adulthood.^19 20^ Therefore, earlier interventions are needed to prevent people from starting to smoke when they are young and reduce future adult smoking rates. ASSIST (A Stop Smoking In Schools Trial) is a ‘peer-led’, school-based smoking prevention intervention, developed in the UK and shown to be effective in preventing adolescent smoking uptake^21^ when the UK had insufficient tobacco control measures, and high rates of smoking, which were rising in females^22^—similar to the current situation in the study sites. ASSIST is based on ‘diffusion of innovation’ theory, with new norms and behaviours promoted through: (1) peer modelling by locally influential individuals; and (2) information disseminated by them through their social networks. ASSIST became a licensed programme in 2010 and has been made widely available via a not-for-profit company, ‘Evidence-to-Impact Ltd’ (https://evidencetoimpact.com/).

## Aims and objectives

The primary aim of the study is to assess the feasibility of conducting a full-scale effectiveness evaluation of an adapted version of the ASSIST intervention in one or more of the three LMICs. As originally planned, the study was to take place in China, Indonesia and the Philippines. However, due to issues with obtaining relevant approvals, China was removed from the trial.

Secondary objectives are to assess, for each country:

1. What, if any, *adaptations to ASSIST* as previously delivered in the UK are required for its delivery in these countries given the different cultural, political, social and educational systems?
2. Is the intervention *feasible and acceptable* to peer supporters, participants and key stakeholders (school staff, education policymakers and parents)?
3. What is the *fidelity and reach* of intervention delivery by trainers and peer supporters, including barriers to, and facilitators of, successful implementation?
4. What is the density, breadth and strength of links within the social networks of each year group, and does this differ between the countries?
5. Does the clustering of smoking within social networks differ across the countries?
6. To what extent are smokers ‘popular’ within each year group, and does this vary by country?
7. Does peer supporters’ ability to reach the whole year group depend on characteristics of the peer supporter, the network structure or the country?
8. What is the reach of peer supporters to ‘at risk’ students?
9. Do the social ties of peer supporters change over the course of the intervention?
10. Are there cross-country differences in the mechanisms of peer selection and influence on smoking?
11. Are we able to recruit and retain schools and peer supporters?
12. Is the evaluation design feasible and acceptable?
13. How applicable are ‘western’ or higher-income country exhaled-breath carbon monoxide (eCO) values in determining active smoking within these countries (given that populations are likely exposed to higher ambient carbon monoxide concentrations from industrial pollution and/or heating and cooking sources of carbon monoxide at home)?
14. Can we collect the required data needed to cost the intervention and conduct a cost-effectiveness analysis?
15. Is there any evidence of social harms (negative social influences, impacts on peer relationships or victimisation) or unintended consequences as a result of the intervention?
16. Is there policy interest in providing (or redirecting) financial support for the intervention to be tested in a future trial and subsequent roll out should a future trial show it to be effective?

## Methods and analysis

This protocol is reported in accordance with the Standard Protocol Item Recommendations for Interventional Trials (SPIRIT) 2013 guidance and outcomes 2022 checklist^23 24^ (online supplemental file 6).

### Trial design

**Figure 1:**
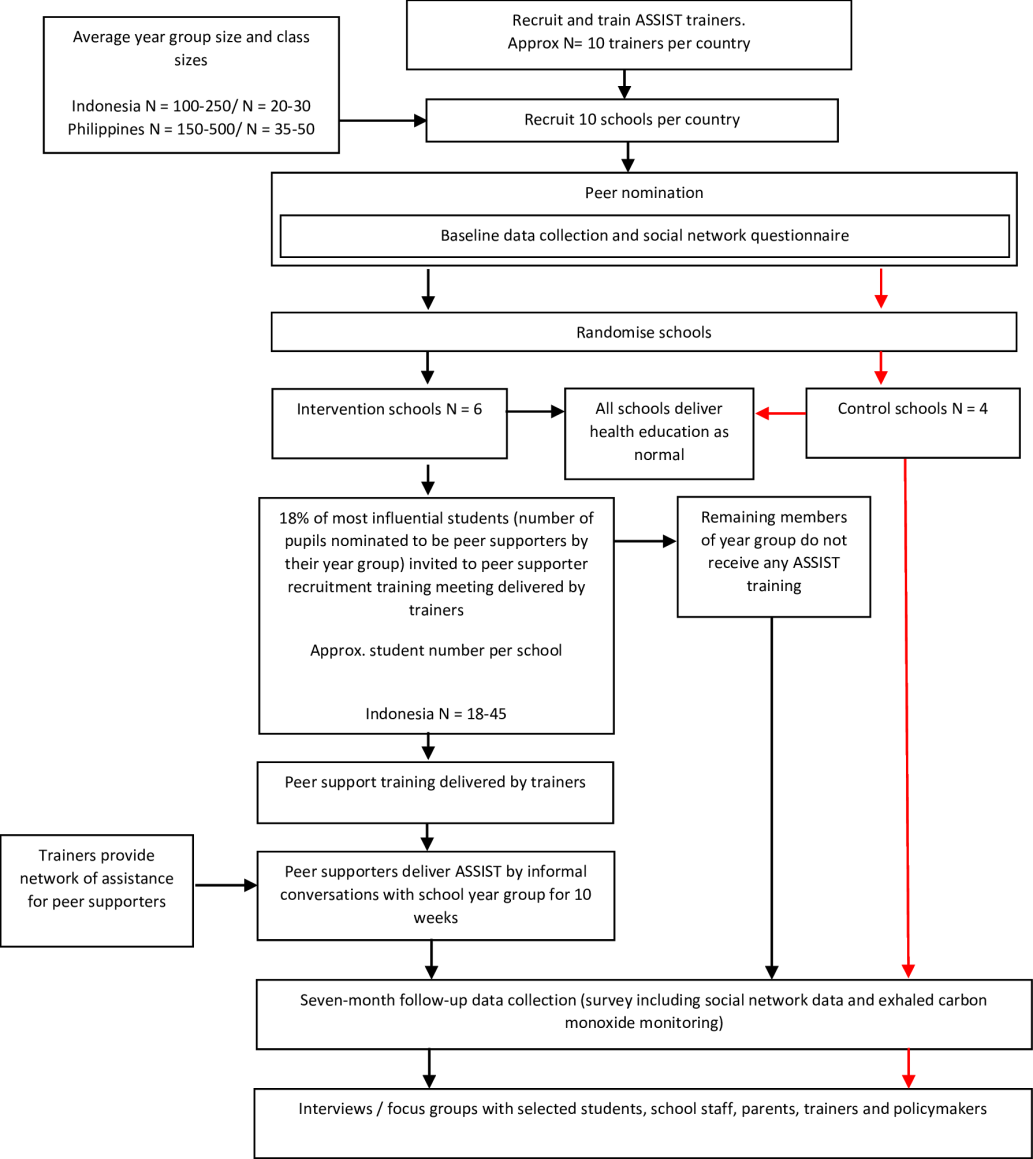
ASSIST global study flow chart. ASSIST, A Stop Smoking In Schools Trial.

The trial is a feasibility cluster randomised controlled trial, with nested process and economic evaluation. Planned start and end dates are 1 March 2022 and 30 December 2025, respectively. The trial will be conducted in 10 schools (six intervention, four control) with between 100 and 500 students per school in each of the two countries (Indonesia, the Philippines). Schools will be randomly allocated to intervention (n=6) or control (n=4). Figure 1 summarises the proposed study flow.

### Trial setting

Private and public schools in the Philippines and Indonesia located in Yogyakarta City and Magelang District in Indonesia; and in and around Manila city in the Philippines.

### Trial participants

Ten secondary schools will be recruited in each country. Depending on the availability of relevant data, we will attempt to specifically target schools in areas of lower socioeconomic status and with high smoking rates. Each year group size will be between 100 and 500 students (mean c.333), so there will be approximately 4000 students in intervention schools and 2666 students in control schools overall. ASSIST is a whole school year-group intervention, thus all students in the target school year (aged 13–14 years) in each country will be potentially eligible to participate in the intervention and research. Students in the target school year who are outside of the 13–14 age group will not be excluded, as students in the target class in Indonesia may be age 15 by public school regulation, and some students in the Philippines may have had to pause or delay their studies due to socioeconomic hardship or challenges in completing their schoolwork.

#### Inclusion criteria

- State and privately funded schools.
- All students in the target year group who are enrolled for school (whether smokers, non-smokers, or experience of smoking).

#### Exclusion criteria

##### Schools

- Schools with school year groups smaller than 100 or greater than 500 will be excluded.
- Schools with limited or poor internet if the study is required to run remotely if COVID situations dictate.
- No single-sex schools (due to the low rates of girls smoking in some countries and also the important influence of the opposite sex on smoking behaviour).

##### Individual students

- Any student identified by the researchers, in consultation with teaching staff, as not having the mental and emotional capacity to consent and fully engage in the research will not be excluded. If a change in mental or emotional capacity occurs during the study, making it difficult for students to take part, then they can be withdrawn from the research process on advice from teaching staff.
- Other reasons for student ineligibility to consent may include learning difficulties, level of literacy and language comprehension issues.

### Randomisation

We will recruit schools for data collection and use cluster randomisation at the school level, with schools being randomised to the intervention or control arms (six intervention and four control) in each country after we have collected their baseline data. Stratification by school year size and a measure of a school’s overall academic performance will be used to reduce the potential for any sizeable differences between intervention and control arm baseline characteristics. Randomisation will be conducted by a member of staff at the University of Glasgow who is independent of the national study teams.

In the Philippines, schools will be stratified by their ‘grade 8 year group size’ and by their NAT score (National Achievement Test; a standardised set of examinations taken by all students). Schools will then be paired into four groups of two to three schools, and within each group, one school will be randomised into the control group, with the remaining schools in each group being assigned to the intervention group.

In Indonesia, a similar process will be followed, but two different measures of school’s academic performance will be used due to differences in how the Yogyakarta City and Magelang Regency regions measure the academic performance of schools. Students in Yogyakarta City undertake a set of standardised examinations known as ‘ASPDs’, whereas in Magelang Regency, the school authorities are not sharing exact academic performances of schools and instead will provide subjective rankings for the five schools in the region taking part in the trial. These subjective rankings will be based on overall academic and non-academic (sports and arts) achievement reports submitted to them by schools. In each region, the five schools will be stratified by school size and by the relevant academic performance measure into two groups of two to three schools, with one school in each group being randomised to the control group and the remaining schools in each group being assigned to the intervention group.

### Consent

**Figure 2:**
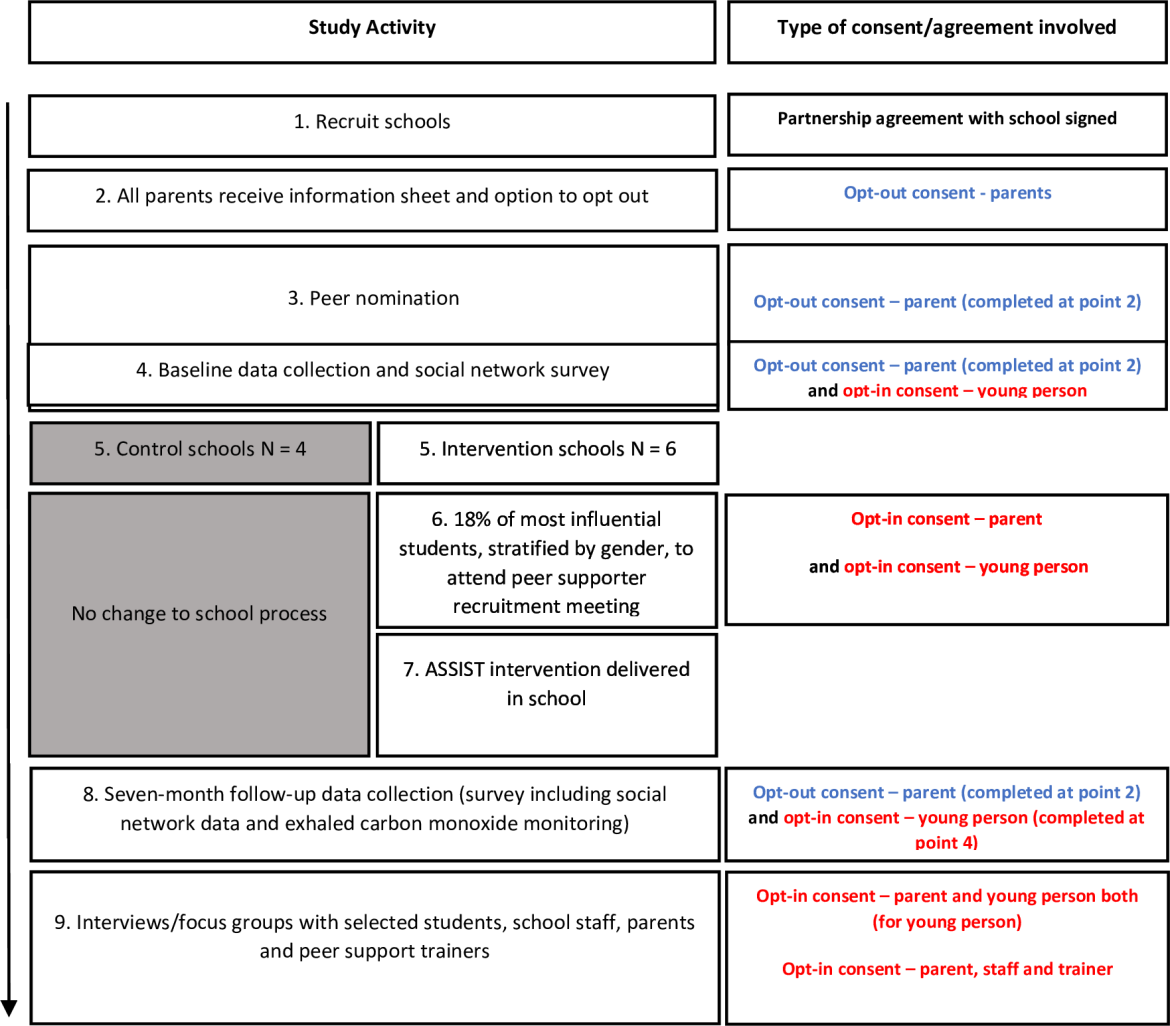
ASSIST global consent flow diagram. Blue, opt-out consent; Red, opt-in consent. ASSIST, A Stop Smoking In Schools Trial.

Consent to participate in the study will be collected from schools, parents, students and trainers (see figure 2 for summary of who provides consent and when at each stage of the study). Initial consent will be obtained from schools to participate in the ASSIST global project by signing a Partnership Agreement. Schools will then disseminate information about the study to parents so they can decide whether to allow their children to participate in the study. Parents will be given a form with the information sheet which they can complete if they wish for their child not to participate in the study (opt out consent) (online supplemental file 1). Students whose parents do not return the slip will be invited to participate in the study using an opt-in consent procedure (ie, the students actively decide to take part) (online supplemental file 2).

### The intervention group

In the initial stages of the study, we will conduct stakeholder work with policymakers, principals, teachers and students to refine the intervention to allow tailoring to the country contexts. We will also recruit trainers who will be trained to deliver the intervention. The training will be piloted in one school, and the trainers will receive feedback.

Schools in the intervention arm will receive the ASSIST intervention in addition to the usual school education on smoking. Peer supporters will be recruited from among the target year group who have been voted as ‘most influential’ by their year group via a short questionnaire. Around 18% of the students who receive the most nominations, stratified by gender, will be invited to a recruitment meeting. Those who choose to take on the role will attend a 2 day training course run by the trainers. Over a defined period of 10 weeks, peer supporters will use social media and face-to-face interaction to disseminate messages aimed at preventing smoking. They will encourage their friends/peers in their year group not to take up smoking. They will be supported—through regular meetings and via social media—by the professionals who trained them. They will also have access to ASSIST global resources (assistglobal.net which is adapted for each country and a moderated forum) for further support. Although only about 18% of the cohort will be trained to be peer supporters, the effect of the programme is likely to be seen across the year group as the remaining students will receive the intervention via informal interactions initiated by the peer supporters.

**Figure 3:**
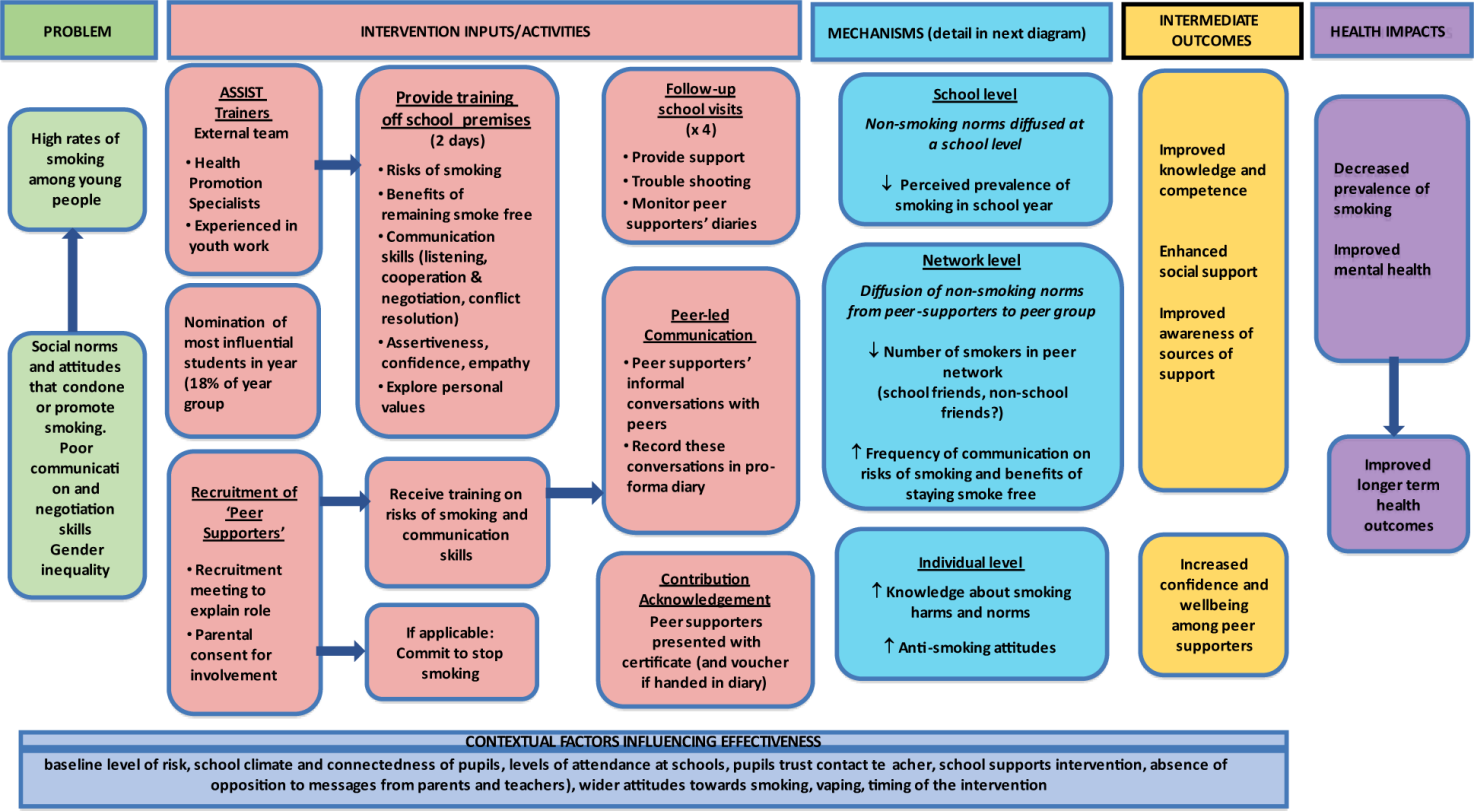
ASSIST global logic model. ASSIST, A Stop Smoking In Schools Trial.

The ASSIST global programme theory is summarised in the logic model below (figures3  4) and was adapted from the STASH study.^25^

**Figure 4:**
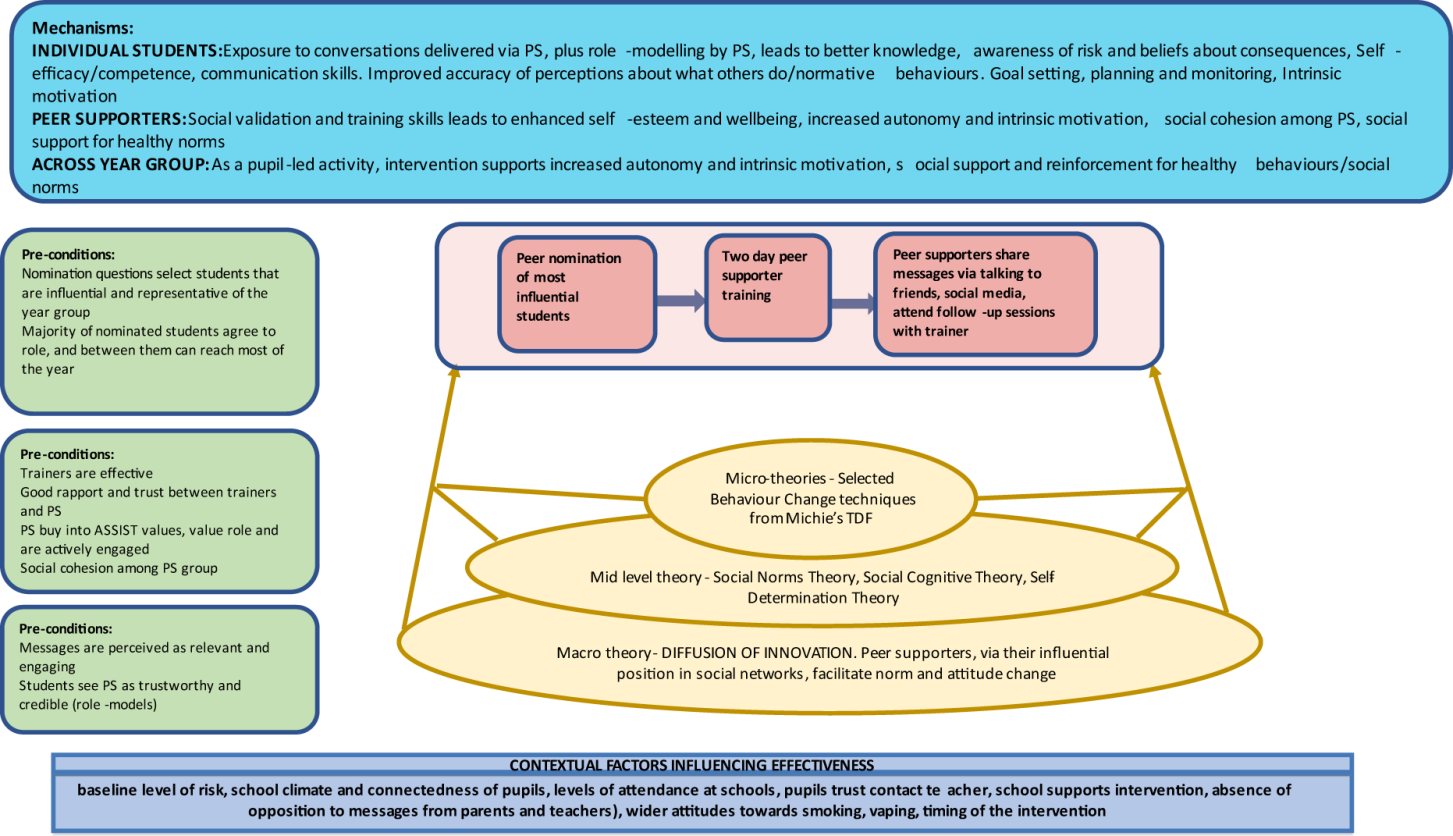
ASSIST global logic emodel (continued). ASSIST, A Stop Smoking In Schools Trial.

### The control group

Control schools in each country will continue with their usual education around smoking.

School-based smoking prevention programmes in Indonesia use the School Health Unit (Usaha Kesehatan Sekolah/UKS) to improve students’ overall health status through the implementation of the Trias UKS. UKS aims to reduce youth smoking rates by increasing students’ awareness of the dangers of smoking and vaping; creating a supportive school environment through regulations and health promotion efforts including no-smoking policies for students and the installation of educational posters and providing smoking prevention and cessation support for students who have already started smoking.^26^ However, the implementation of smoking prevention initiatives through health promotion, as mandated by government regulations, remains suboptimal. According to the Global Youth Tobacco Survey (GYTS) 2009, only 69.3% of students received information about the dangers of smoking at school.^5^

In the Philippines, the Department of Health implements programmes designed to inform the population about non-communicable diseases and target substance abuse, which are accessible through residential communities and publicly funded schools.^27^ It also created the Adolescent Youth Health subprogramme composed of government and non-government units working on addressing health issues specific to adolescents, with the overarching goal to improve the health status of adolescents through provision of prevention services that focus on health education and building life skills—creating a system that increases knowledge and changes behaviour that leads to the avoidance of risky behaviours and increases practice of protective behaviours, and utilisation of services.^28 29^ The Department also implements Barkada Kontra Droga, a programme intended to dissuade young people from substance use, promoting a drug-free and productive life through activities such as youth camps, seminars and competitions.^30^

The activities vary by country and also across schools, but we will capture details of this as part of the process evaluation. At the end of the study, we will offer something to the control group schools such as resources and talks by the study team.

### Retention of participants

On receipt of the signed Partnership Agreement, the school will be considered formally recruited to the study. Any school opting to discontinue with the study after this point will be considered a withdrawal. To maximise retention, schools will be given compensation for disruption to school life due to the questionnaire and process evaluation activities.

Given the whole year-group nature of ASSIST, individual opt-out from the intervention will not be possible. However, those nominated as influential and invited to become peer supporters have the right to withdraw consent for this at any time, as do all those participating in any aspect of the research process. If a participant initially consents but subsequently withdraws from the study, a clear distinction will be made as to what aspect of the study the participant is withdrawing from.

Leaflets detailing the benefits of becoming a peer supporter have been developed to maximise parental consent for students to sign up as peer supporters. Small reimbursements (gifts/vouchers) will be provided to those involved in the data collection as well as research interviews/focus groups (school staff, students and parents). This will vary by country; drinks and healthy snacks may also be provided during group consultation or research sessions.

### Outcome measures and progression criteria

The primary outcome of this feasibility trial will be to assess whether prespecified progression criteria are met to progress to a full effectiveness trial of the ASSIST intervention. The country-level progression criteria are described in online supplemental table 1. These have been approved by our independent Trial Steering Committee (TSC). Final assessment of the progression criteria will be undertaken by the independent TSC in collaboration with the Trial Management Group (TMG), following analysis of the findings.

Outcome data will be collected at baseline (prerandomisation) and 7 month follow-up postrandomisation (see table 1 for an overview of outcome measures at each timepoint). In-country researchers will distribute questionnaires to the whole year group in all schools. At baseline, this will be completed after the peer nomination, which will happen on the same day in all schools. The baseline questionnaire (onlinesupplemental files 3  4) will gather the outcomes listed in table 1 as well as additional data known to be related to smoking. At the 7 month follow-up, we will repeat the same questionnaires but also use a standard objective method of assessing recent active smoking: eCO using a Smokerlyzer Micro+device.

**Table 1:** Outcome measures and effect modifiers

Outcome/effect modifier	Timepoint	Measured by
Smoking behaviour (own and that of friends and family including spillover effects)	Baseline and 7 months	Study developed questionnaire
e-cig/vaping and other forms of tobacco use	Baseline and 7 months	Study developed questionnaire
Smoking-related attitudes and knowledge	Baseline and 7 months	Study developed questionnaire
Smoking norms	Baseline and 7 months	Study developed questionnaire
self-esteem	Baseline and 7 months	Study developed questionnaire
Sociodemographics	Baseline only	Study developed questionnaire
eCO	7 months only	Smokerlyzer Micro+

eCO, exhaled-breath carbon monoxide.

The primary effectiveness outcome is self-reported weekly smoking (ie, those reporting they smoke 1+cigarettes weekly) measured via the study questionnaire at baseline and 7 month follow-up. Informed by our programme theory, we will investigate a range of secondary outcomes and effect modifiers as listed above. The study questionnaire is based on the original ASSIST trial questionnaire with adaptations (ie, socioeconomic questions tailored to be locally relevant) and updates (eg, vaping questions).^21 31^

We will also do some exploratory work looking at concentration thresholds for eCO to examine the level of agreement between self-reported smoking and measured eCO concentrations. The traditional ‘cut-points’ for identifying smoking (0–6 ppm: non-smoker; 7–9: borderline; 10+: active smoking) have been developed in ‘western’ countries where outdoor air concentrations of CO tend to be low and where indoor combustion of fuels (eg, coal, wood and kerosene) is uncommon and rarely leads to high exposure. In countries where outdoor and indoor concentrations of CO are higher, it is possible that non-smoking children will have eCO levels that exceed the standard indicative values for active smoking.^32^

There is a possibility that we will identify high readings of carbon monoxide on the Smokelyser device even when children claim that they are not smoking. This may be caused by exposure to poorly ventilated or simple cooking/heating systems. In this case, we will give the children a letter to take home to their parents advising them to check the safety of household heating and/or cooking arrangements. Outcome data will be collected from all participants who discontinue or deviate from the intervention protocol as long as they are willing.

### Process and health economic evaluation

As part of the trial, a process evaluation will be undertaken to determine the feasibility, fidelity, acceptability and reach of the intervention in these novel contexts. We will also record the key costs of the intervention and assess the feasibility of measuring outcomes including health-related healthcare resource use.

A process evaluation framework will be developed at the start of the study and will cover the:

- *Feasibility and acceptability of intervention*: for example, the proportion of nominated peer supporters who agreed to participate, the acceptability of the intervention to teachers and parents.
- *Feasibility and acceptability of the study processes* to peer supporters, non-peer supporters, teachers and parents: that is, recruitment, retention and data collection.
- *Reach of the intervention*: we will assess the level of exposure to the intervention via the social network questionnaire. We will also explore this in the interviews/focus group.
- *Fidelity of implementation*: we will assess whether the key components of the ASSIST intervention were delivered, and what components, if any, were not delivered with fidelity.
- *Contextual factors and programme theory*: we will assess contextual factors at individual-level, school-level and country-level and apply these to a refined programme theory. We will also explore the wider policy environment.
- *Impact* of the study/intervention on peer supporters, including changes in the confidence, self-esteem or social networks of the peer supporters.
- *Contamination*: that is, where students in control schools hear about ASSIST or receive elements of ASSIST (eg, via social media) as well as what control and intervention schools are delivering to students regarding smoking prevention.

Process data will be gathered using multiple methods including questionnaires, observations, interviews and focus groups as well as social network data. The follow-up questionnaire will include items for: non-peer supporters to assess awareness, understandings and opinions of the intervention; and for peer supporters, we have a short questionnaire which will assess their understandings and opinions of the intervention. We will also ask peer supporters about how much they engaged with the intervention, for example, number of conversations, messages posted, etc. Students in all schools will also be asked about conversations about smoking and key smoking-related knowledge items in the questionnaire.

#### Observations

Observations of peer supporter nomination and recruitment, peer supporter training and peer supporter follow-up sessions will be conducted by research staff. Peer supporter training will be filmed for Evidence-to-Impact (the training provider) to quality assure intervention delivery, and this data will be used for the intervention fidelity assessment.

#### Qualitative data

Qualitative interviews and focus groups will explore views and experiences of feasibility, acceptability, adaptability to the country context, barriers, facilitators and reach of the intervention and its components and potential adaptations. Comparison between countries and within and across different participant groups involved in the intervention, such as trainers’ views and peer supporters’ views, will be enabled through a Framework Analysis approach.

In intervention schools, we will aim for: interviews with two staff members per school (n=12), three parents per school (n=18) and three student focus groups per school (two with peer supporters, one with non-peer supporters). Peer supporter focus groups will have a minimum of six students per group (n=72 peer supporters per country). Non-peer supporter focus groups will have a minimum of 8 per group (n=48). We may also conduct paired student interviews if focus groups prove challenging to organise. In control schools, we will interview one staff member per school (n=4). We will also interview up to six ASSIST trainers per country and four policy makers per country. See online supplemental file 5 for the interview guides.

#### Social network data

We will collect sociocentric data on friendship networks at baseline and follow-up. We will use this data to investigate the patterns of social structure within each school and assess the network position of peer supporters (eg, are they well connected or central in the network?). Data will be collected about friendships within the year group, as well as out-of-school friends.

#### Web/app analytics

We will collect website usage data for both peer supporters and non-peer supporters using Google Analytics to assess online engagement with the intervention.

#### Health economics data

We will develop a data collection form to pilot collection of the key resources used in the intervention implementation and maintenance. We will assess the completeness of the data collected, highlighting any missing data.

#### Sample size and power

The study does not aim, and is not designed, to identify a precise estimate of the effectiveness of the intervention within any one country or across countries. To do so would require longer follow-up than the 7 months, we propose and a sample size in the approximate magnitude (c. 60 schools and 12 000 students) of the original ASSIST trial.^33 34^

#### Quantitative analysis

A detailed Statistical Analysis Plan has been drafted and will be finalised before the data collection is complete.

##### Baseline characteristics

Baseline characteristics will be summarised overall and by country and intervention group, with statistical comparison of baseline data being made between countries. These baseline characteristics include sociodemographic information such as age, gender and ethnicity, as well as information related to smoking that will be analysed as secondary outcomes during the analyses.

##### Feasibility outcomes

These will be the primary focus of the analysis. The assessment of whether and how feasible it is to conduct a full-scale effectiveness evaluation of an adapted version of the ASSIST intervention in Indonesia and the Philippines will be done through descriptive statistics for each country for each of the quantitative progression criteria.

##### Efficacy outcomes

To identify effect estimates, a cluster-level analysis will be undertaken with school-level strata, baseline weekly smoking and gender included as covariates. Country will be included as a fixed effect in a model combining data from both countries, and country-specific models will also be estimated. Results will be reported in the form of risk ratios and risk differences, with corresponding 95% CIs. The primary analysis will be of risk ratios in separate country-specific models.

The primary effectiveness outcome is self-reported weekly smoking. Secondary outcomes will include student-level variables such as smoking behaviour (own and that of friends and family); e-cig use and vaping and other forms of nicotine use; smoking-related attitudes and knowledge; smoking norms; self-esteem; spillover effects on parents, etc, and self-efficacy. These analyses will be exploratory as the study is not powered to identify precise estimates of effect.

Missing data will not be imputed, unless more than 20% of cases are lost due to missing data for the primary outcome at follow-up, in which case multiple imputation will be performed. Sensitivity analysis will also be carried out in the event of multiple imputation to compare the effect of missing data on the primary outcome and compare alternative imputation methods.

##### Social network analysis

Social network analysis will be used to assess intervention acceptability and feasibility—that is, whether peer supporters reached most of the year group. Analyses will identify the percentage of friendship clusters that have at least one peer supporter within them. Clustering algorithms (eg, Girvan-Newman) will be used for these analyses.

Social network analysis will also be used in exploratory analyses. Descriptive analyses will measure the structure of each network, such as how densely connected the friendship clusters are and compare these across countries. The tendency for students to be friends with peers who share their smoking habits will be tested, as well as the extent to which smokers are popular in the network. Longitudinal network models (eg, Stochastic Actor-Oriented Models) will measure changes in friendships and smoking over the course of the intervention. We will also measure the reach of peer supporters to ‘at-risk’ students (students who smoke), to determine if the supporters reached those most in need of the intervention.

##### Health economic analysis

The health economic analysis aims to evaluate the feasibility and acceptability of acquiring resource utilisation data to estimate the costs associated with the implementation of the ASSIST intervention in Indonesia and the Philippines. The availability of country-level data on the financial implications of smoking will be assessed, including the impact on health services and employment. The feasibility of collecting unit costs from each country will also be evaluated. The following will be estimated: the intervention cost per pupil within each school; the intervention cost in each country and an incremental cost per student not smoking. The feasibility of estimating the impact on the environment resulting from smoking will be assessed using publicly available data combined with the primary outcome of self-reported weekly smoking.

### Qualitative analysis

#### Observations

Video-recordings of student training sessions will be graded against an observation checklist to determine fidelity to the intervention delivery.

#### Interviews and focus groups

A thematic framework method will be used to analyse the qualitative data.^35 36^ This approach typically uses a seven-step process. We will complete the following stages (step 1 is an additional step).

1. Development of preliminary coding framework aligned to the research questions and informed by research on smoking interventions in adolescents.
2. Transcription and translation of the data.
3. Data familiarisation.
4. Preliminary coding of initial transcripts
5. Refinement of the coding framework based on the preliminary coding
6. Application of the coding framework to all the data
7. Charting and summarising the data into framework matrices. Thematic categories will be largely predetermined, but emergent themes will also be added to the matrices. Each country will have its own matrix to enable within and between country comparisons.
8. Interpretation of the data.

Data will therefore be coded both inductively and deductively.^37^ Around 20% of transcripts from each country will be professionally translated and checked by the local study team before being transferred for review and double coding by the UK team. In addition, country teams will double code an additional 20% of the interviews within their teams.

### Mixed methods

Qualitative and quantitative data are being collected and analysed separately using a convergent parallel approach to mixed-methods research.^38^ The qualitative data, which brings greater depth, are key to understanding the feasibility and acceptability questions. Bringing together both data sets in the interpretation stage provides an opportunity to triangulate the data to offer further insights.

### Patient and public involvement

A Stakeholder Advisory Group (SAG) and a Youth Advisory Group (YAG) have been established in each country. The SAGs include 5–7 members who are public health/education officials, school principals/teachers, academics, parents; and the YAGs are made up of 5–6 students of the same target age group from schools that are not involved in the study.

The SAG and YAG will contribute to the design of the feasibility trial, including adaptations to the intervention and study materials (eg, study questionnaire, training activities in the manual and relevant resources such as posters), as relevant to the local culture and context. The SAG and YAG will be involved throughout the duration of the study and dissemination of findings. The process of deciding on adaptations will involve consultation with partners and our advisory groups. The trial team will make final decisions on adaptations considering both what is feasible to implement and also what can be adapted while retaining the key intervention mechanisms described in the programme theory.

## Trial management

The TMG comprising the Principal Investigator, coinvestigators and Project Manager will meet regularly and review progress and important protocol modifications. The TMG will report to the independent TSC which consists of a chair, a statistician and other experts, and will operate according to Medical Research Council guidance (https://www.ukri.org/publications/mrc-short-guide-to-trial-steering-committees/). Routine reports reviewed by the TSC will include a summary of adverse events if there are any. The TSC will cover the functions of the Data Monitoring and Ethics Committee (DMEC) due to the low-risk nature of this study and the lack of a need for unblinded/interim analysis.

### Assessment of harms

ASSIST is a low-risk intervention and therefore we do not expect any serious adverse events to occur. We have delivered earlier versions and adapted forms of this intervention in a number of studies, and there have been very few incidences of harm. We have identified possible risks and have taken steps to mitigate against these. These steps and the reporting process are detailed in our Safeguarding Policy.

## Data management

The study team will adhere to the Data Management Plan for safe and accurate data management. All study data will be gathered by trained researchers using hardcopy paper questionnaires or via the Research Electronic Data Capture (REDCap) database. Data will be pseudonymised and stored securely.

Qualitative data will be collected on encrypted audio recording devices and transferred to secured university drives in each country. Audio files will be transcribed and deidentified. A portion of transcripts will be translated and shared with the UK team for analysis. A between-country data sharing agreement will be implemented, and qualitative and quantitative data will be shared using the University of Glasgow service (transfer.gla.ac.uk).

Personal contact data, personal data and research data will be securely stored locally in accordance with local country regulations. All data will be kept for 10 years in line with University of Glasgow Research Governance Framework Regulations.

## Ethics and dissemination

The trial has been approved by the University of Glasgow College of Medical, Veterinary and Life Sciences (MVLS) Ethics Committee (ref: 200210204), the De La Salle University Research Ethics Review Committee (ref: 2023-012C) and the Medical and Health Research Ethics Committee (MHREC); Faculty of Medicine, Public Health and Nursing; Universitas Gadjah Mada (ref: KE/FK/1205/EC/2022). The trial is sponsored by the University of Glasgow (Head of Research Regulation and Compliance—debra.stuart@glasgow.ac.uk). The sponsor will not have input in data collection, management, analysis and interpretation; write up and submissions for publication.

A publication policy for the study will be developed, and coauthors and team members will be offered authorship on the study papers.

The study findings will be disseminated through peer-reviewed publications in expert journals and conference presentations and targeted communications to schools, policymakers and the public.

## Supplementary material

10.1136/bmjopen-2024-096963 online supplemental file 1

10.1136/bmjopen-2024-096963 online supplemental file 2

10.1136/bmjopen-2024-096963 online supplemental file 3

10.1136/bmjopen-2024-096963 online supplemental file 4

10.1136/bmjopen-2024-096963 online supplemental file 5

10.1136/bmjopen-2024-096963 online supplemental file 6

10.1136/bmjopen-2024-096963 online supplemental table 1
