# Supplementary material for: A Stop Smoking In Schools Trial in three culturally different middle-income countries (ASSIST global): protocol for a randomised feasibility study
Source: BMJ Open. 2025 Jun 22;15(6):e096963. doi: 10.1136/bmjopen-2024-096963 (PMC12184387; doi:10.1136/bmjopen-2024-096963)
Supplement: online supplemental file 1 [file bmjopen-15-6-s001.docx]

**Consent form for Parents/Guardians study awareness**

**PLEASE ONLY COMPLETE AND RETURN THIS FORM IF YOU DO NOT WANT YOUR CHILD TO TAKE PART**

Project Number: 308538

Title of Project: ASSIST Global

Name of Researcher(s):

Principal investigator - Professor Sharon Simpson

Co-investigators - Professor Laurence Moore, Dr Kate Reid, Dr Sean Semple, (add country research team)

If you **DO NOT want your child to take part** then please initial the box, sign below and return this form to the school/research team.

I DO NOT give my permission for my child to take part in the study.

- By signing, I confirm that I have read and understood the ASSIST Global Information Sheet for Parents/Guardians study awareness (V3.0 06.11.2023) and I understand what the research is about.
- I also confirm that I have no questions to ask or have asked questions to the research team/teacher in the school and have decided not to allow my child to take part.

|  |  |  |
| --- | --- | --- |
| Your name (please print) |  | Your child’s name (please print) |
|  |  |  |
| Your signature |  | Today’s date |

**PLEASE NOTE THAT IF YOU ARE HAPPY FOR YOUR CHILD TO TAKE PART THEN PLEASE DON’T RETURN THIS FORM.**

**THANK YOU**
