# Supplementary material for: A Stop Smoking In Schools Trial in three culturally different middle-income countries (ASSIST global): protocol for a randomised feasibility study
Source: BMJ Open. 2025 Jun 22;15(6):e096963. doi: 10.1136/bmjopen-2024-096963 (PMC12184387; doi:10.1136/bmjopen-2024-096963)
Supplement: online supplemental file 2 [file bmjopen-15-6-s002.docx]

Centre Number:

Project Number: 308538

Participant Identification Number for this trial:

**Title of Project: ASSIST Global**

**Name of Researcher(s):**

Principal investigator - Professor Sharon Simpson

Co-investigators - Professor Laurence Moore, Dr Kate Reid, Dr Sean Semple, (add country research team)

**Consent form for young person baseline and follow-up data collection**

1. I confirm that I have read the baseline and follow-up data collection information sheet (V3.0 06.11.2022) and privacy notice (V2.0 06.11.2023) and have had the opportunity to ask questions.
2. I understand that my participation is voluntary and that I can end my participation at any time without giving a reason, and without my legal rights being affected, and that data collected until the point of withdrawal will be retained.
3. I agree to take part in this aspect of the study.

**If you do not agree with the following statement this will not affect your participation in the study:**

I agree to my data being deposited in a data archive and, where relevant,

shared with trusted researchers or educators (some from outside my country) Yes No

in a form that makes it impossible for them to identify me.

#### Name of participant Date Signature

#### Name of Person taking consent Date Signature

(if different from researcher)

#### Researcher Date Signature
