# Supplementary material for: A Stop Smoking In Schools Trial in three culturally different middle-income countries (ASSIST global): protocol for a randomised feasibility study
Source: BMJ Open. 2025 Jun 22;15(6):e096963. doi: 10.1136/bmjopen-2024-096963 (PMC12184387; doi:10.1136/bmjopen-2024-096963)
Supplement: online supplemental file 3 [file bmjopen-15-6-s003.docx]

YOUNG PEOPLE’S VIEWS ON SMOKING QUESTIONNAIRE

The questionnaire is completely confidential, anything you put in the survey is kept secret by the ASSIST Global research team. We will keep your data securely and only use it for research purposes. Anonymous data may be shared with genuine researchers. Anonymous data means that we will delete all names and any other personal details so nobody can find out about your answers

Your friends, family, teachers and school will not find out your answers.

Take your time to read the questions carefully and please answer them as accurately and honestly as you can. If you are unsure what a question means, please ask one of the researchers for help. Please remember that this is NOT a test. There are no right or wrong answers!

If you are completing a paper questionnaire there is no need to write your name on the questionnaire.

After you have filled it in, you can put it in the envelope we’ve given you and seal it.

Most of the questions can be answered by putting a tick in the box next to the answer that applies to you – like this:

| Yes | 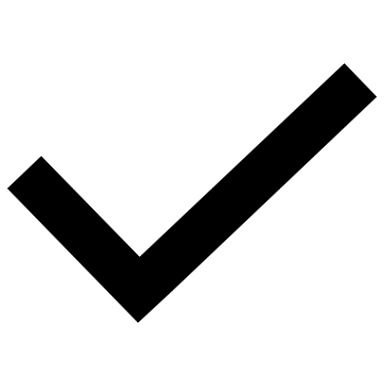 | _1_ |
| --- | --- | --- |
|  |  |  |
| No |  | **_2_** |

Read the instructions carefully for each question.

Some questions don’t apply to everybody. It always tells you by the box that you tick which question to answer next.

#### **SECTION ONE**

#### **First, a few details about yourself.**

#### **Please answer all of the questions on this page.**

1) How would you describe your gender (Please tick **ONE box only**)

Girl 1

Boy 2

I describe myself in another way 3

I prefer not to say 4

2) How old are you? (Please tick **ONE box only**)

12 years old 1

13 years old 2

14 years old 3

3) Which ethnic group do you belong to? (Please tick **ONE box only**)

Filipino 1

Chinese-Filipino 2

Chinese 3

Other 4

#### **SECTION TWO**

#### **This part of the questionnaire is about smoking.**

#### **Remember that your name is not on the questionnaire, so no-one you know will ever find out your answers.**

4) Do any of the following people smoke?

**Yes No I don’t know I don’t have one**

a) Best friend 1 2 3 4

b) Boyfriend/girlfriend (dating) 1 2 3 4

Go to question 5

5) Do your parents/guardians smoke cigarettes now?

**Yes** **No N/A**

Mother/female guardian 1 2 3 Go to question 6

Father/male guardian 1 2 3 Go to question 6

6) Does anyone else (besides your parents/guardians) in your house smoke cigarettes now?

Yes 1 Go to question 6a

No 2 Go to question 7

**6a) If you have ticked ‘Yes’, please say who they are below:**

Brother 1

Sister 2

Grandfather 3

Grandmother 4

Uncle 5

Aunt 6

Other 7

If you have ticked “Other’, please write who they are: ____________________________________

Go to question 7

7) Does anyone **who lives in your house** use other kinds of tobacco e.g. vapes, smokeless tobacco? (Please don’t include yourself).

Yes 1 Go to question 7a

No 2 Go to question 8

**7a) If you have ticked ‘yes’, please say who they are below:**

Father 1

Mother 2

Brother 3

Sister 4

Grandfather 5

Grandmother 6

Uncle 7

Aunt 8

Other 9

If you have ticked “Other’, please write who they are: ____________________________________

Go to question 8

8) Now read all of these statements carefully and tick the box next to the one which describes you best (Please tick **ONE box only**).

I have never smoked 1 Go to question 9

I have only tried smoking once 2 Go to question 9

I used to smoke sometimes but I never smoke a cigarette now 3 Go to question 14

I sometimes smoke cigarettes now but I smoke less than one a week 4 Go to question 11

I usually smoke between one and six cigarettes a week 5 Go to question 10

I usually smoke more than six cigarettes a week 6 Go to question 10

9) Just to check, read these statements carefully and tick the box next to the one which describes you best (Please tick **ONE box only**).

I have never tried smoking a cigarette, not even a puff or two 1 Go to question 22

I once tried a puff or two of a cigarette, but I never smoke now 2 Go to question 14

I sometimes smoke cigarettes 3 Go to question 11

10) When did you start smoking at least one cigarette a week?

Less than 3 months ago 1 Go to question 11

3 to 6 months ago 2 Go to question 11

7 months to 1 year ago 3 Go to question 11

More than one year ago 4 Go to question 11

11) How do your parents (or guardian) feel about you smoking? (Please tick **ONE box only**).

They don’t like it 1 Go to question 12

They don’t mind 2 Go to question 12

They don’t know I smoke 3 Go to question 12

I don’t know 4 Go to question 12

12) Where do you get your cigarettes from? (Please tick Yes or No for **EACH** line).

**Yes** **No**

a) I buy them from a convenience store 1 2

b) I buy them from a vending machine 1 2

c) I buy them from friends 1 2

d) I buy them from relatives 1 2

e) I buy them from people at school 1 2

f) Friends give them to me 1 2

g) My brother or sister gives them to me 1 2

h) My mother or father gives them to me 1 2

i) People at school give them to me 1 2

j) I get them in some other way 1 2

Go to question 13

13) When do you smoke? (Please tick Yes or No for **EACH** line).

**Yes No**

a) When you are with friends 1 2

b) When you are on your own 1 2

c) During school breaks 1 2

d) On the way to or from school 1 2

e) With your family 1 2

f) At home 1 2

g) Only at weekends 1 2

Go to question 14

14) How old were you when you first tried smoking a cigarette, even if it was only a puff or two? **Please write your answer in the box.**

I was years old Go to question 15

15) How long is it since you last smoked a cigarette (even if it was only a puff or two)?

(Please tick **ONE box only**).

Less than one day 1 Go to question 16

One to three days 2 Go to question 16

Four to seven days 3 Go to question 16

More than a week but less than a month 4 Go to question 16

One to two months 5 Go to question 16

Three to six months 6 Go to question 16

More than six months 7 Go to question 16

16) If you are a smoker, have you tried to quit?

Yes 1 Go to question 17

No 2 Go to question 22

17) How many times? _____________

1 1 Go to question 18

2-4 2 Go to question 18

5 and more 3 Go to question 18

18) When did you last try to quit? ____________

In the last week 1 Go to question 19

2-4 weeks ago 2 Go to question 19

2-3 months ago 3 Go to question 19

4 or more months ago 4 Go to question 19

**Yes No**

19) If you are a smoker, do you plan to give up smoking? 1 2

Go to question 20

**Yes No**

20) Are you aware of any websites that give information 1 2

about smoking or stopping smoking?

Go to question 21

**Yes No**

21) Do you know where to access help or support to stop smoking? 1 2

Go to question 22

22) Do you use e-cigarettes or vape?

Yes 1 Go to question 23

No 2 Go to question 23

23) Do you use smokeless tobacco (e.g., nicotine bags)?

Yes 1 Go to question 24

No 2 Go to question 24

24) Please read the following statements. Please think about whether they are either true or false.

(Please mark an **X in one box on every line**)

**True False**

a) Smoking makes teeth yellow 1 2

b) Smoking causes wrinkles 1 2

c) Smoking makes smokers smell bad 1 2

d) Smoking causes shortness of breath 1 2

e) Smoking causes diarrhoea 1 2

f) Smoking causes bad breath 1 2

g) Smoking causes cough 1 2

h) Smoking leads to increased risk of lung cancer 1 2

i) Smoking leads to greater risk of heart disease 1 2

j) Smoking causes flu 1 2

k) Smoking can lead to tooth loss 1 2

l) One cigarette won’t make me a smoker 1 2

Go to question 25

25) What percentage of **girls in your year group** do you think smoke? **Place a mark on the line which corresponds to the percentage you think smoke - between 0 and 100%.**

0 20% 40% 60% 80% 100%

______________________________________________________________________

26) What percentage of **boys in your year group** do you think smoke? **Place a mark on the line which corresponds to the percentage you think smoke - between 0 and 100%.**

0 20% 40% 60% 80% 100%

______________________________________________________________________

27) What percentage of **adult women** do you think smoke? **Place a mark on the line which corresponds to the percentage you think smoke - between 0 and 100%.**

0 20% 40% 60% 80% 100%

______________________________________________________________________

28) What percentage of **adult men** do you think smoke? **Place a mark on the line which corresponds to the percentage you think smoke - between 0 and 100%.**

0 20% 40% 60% 80% 100%

______________________________________________________________________

29) If you are **not currently** a smoker, how confident are you that you can resist pressure by your friends to smoke? (Please tick **ONE box only**).

Not confident 1 Go to question 30

A little confident 2 Go to question 30

Neutral/don’t know 3 Go to question 30

Somewhat confident 4 Go to question 30

Very confident 5 Go to question 30

30) If you are **not currently** a smoker, how confident are you that you will remain a non-smoker? (Please tick **ONE box only**).

Not confident 1 Go to question 31

A little confident 2 Go to question 31

Neutral/don’t know 3 Go to question 31

Somewhat confident 4 Go to question 31

Very confident 5 Go to question 31

#### **SECTION THREE**

#### **The next set of questions are about your friends and family.**

#### **Please remember that no-one that you know will see**

#### **the answers that you give here.**

31) How many close friends do you have **at school**? (Please tick **ONE box only**).

None 1 Go to question 32

One 2 Go to question 32

Two to three 3 Go to question 32

Four to five 4 Go to question 32

Six or more 5 Go to question 32

**These questions are about time you spend FACE TO FACE (i.e., in person) with your friends:**

32) How often do you spend time with school friends **right after** school?

(Please tick **ONE box only**).

Four to five days a week 1 Go to question 33

Two to three days a week 2 Go to question 33

Once a week or less 3 Go to question 33

Never 4 Go to question 33

I don’t have any friends at school at the moment 5 Go to question 35

33) How many **evenings** per week do you **usually** spend out (away from your home) with your friends from school? (Please **CIRCLE** your answer).

0 1 2 3 4 5 6 7 evenings a week

Go to question 34

34) How often do you spend time at **weekends** with your friends from school?

(Please tick **ONE box only**).

I **often** spend time at weekends with friends **from school** 1 Go to question 35

I **sometimes** spend time at weekends with friends **from school** 2 Go to question 35

I **hardly ever** spend time at weekends with friends **from school** 3 Go to question 35

I **never** spend time at weekends with friends **from school**  4 Go to question 35

**These questions are about how much time you spend ONLINE with your friends (e.g. on Snapchat, Whatsapp, Facebook etc):**

35) What apps do you use to get online and chat to your friends e.g. Facebook/Whatsapp/Line/Twitter/Instagram/TikTok/Viber/other? (Please tick **all that apply**)

Facebook 1 Go to question 36

TikTok 2 Go to question 36

Instagram 3 Go to question 36

Twitter 4 Go to question 36

Discord 5 Go to question 36

Viber 6 Go to question 36

Other 7 **Please specify ________________** Go to question 36

None 8 Go to question 37

36) Which of these statements applies to you? (Please tick **ONE box only**)

**Yes No**

I go online to chat to friends often. 1 2

I have an account with one of the above apps but I don’t often use it to chat with friends. 1 2

Go to question 37

37) How often do you spend time online with friends from school **right after** school?

(Please tick **ONE box only**).

Five days a week 1 Go to question 38

Two to four days a week 2 Go to question 38

Once a week or less 3 Go to question 38

Never 4 Go to question 38

I don’t have any friends at school at the moment 5 Go to question 41

38) How often do you spend time online at **weekends** with your friends from school?

(Please tick **ONE box only**).

I **often** spend time online at weekends with friends **from school** 1 Go to question 39

I **sometimes** spend time online at weekends with friends **from school** 2 Go to question 39

I **hardly ever** spend time online at weekends with friends **from school** 3 Go to question 39

I **never** spend time online at weekends with friends **from school**  4 Go to question 39

39) Do you talk about smoking with your school friends?

Yes 1 No 2 Go to question 40

40) How many conversations have you had with friends **from school** about smoking **in the last week**? Please write the number of times in the box below. If you have not had any conversations about smoking in the last week with friends, please write 0 in the box:

Go to question 41

41) These questions are about your relationship with **all your friends, not just school friends**. For each question, please say how often this is true for you and your friends.

(Please tick **ONE BOX FOR EACH LINE**).

**Never Sometimes Often Almost always I don’t have friends**

a) My friends listen 1 2 3 4 5

to what I have to say

b) I feel my friends are 1 2 3 4 5

good friends

c) I like to get my

friends’ point of view 1 2 3 4 5

on things

d) My friends are

concerned about my 1 2 3 4 5

well-being (i.e., how

I am)

e) I depend on my 1 2 3 4 5

friends for support

Go to question 42

42) Which of these sentences best describes your friends? (Please tick **ONE box only**).

All of my friends smoke 1

Most of my friends smoke 2

Some of my friends smoke 3

None of my friends smoke 4

I don’t have any friends 5

Don’t know/not sure 6

Go to question 43

#### **SECTION FOUR**

#### **This part of the questionnaire is to find out more about**

#### **you and your views on life.**

#### **Please read each statement carefully before giving your answer.**

43) Below are some statements about smoking. How much do you agree or disagree with them? (Please tick **ONE box for each line**).

**Strongly agree Agree Not sure Disagree Strongly disagree**

a) Trying a cigarette 1 2 3 4 5

is part of growing up.

b) People of my age 1 2 3 4 5

who smoke look cool.

c) People like you less 1 2 3 4 5

if you smoke.

d) Smoking keeps you 1 2 3 4 5

slim.

e) People of my age 1 2 3 4 5

smoke to show that

they can do what they like.

f) People who smoke

are less likely to 1 2 3 4 5

feel bored.

g) Smoking helps you to 1 2 3 4 5

make friends.

h) Smoking helps you to 1 2 3 4 5

lose weight.

i) People of my age

who smoke look 1 2 3 4 5

grown up.

j) It is sometimes

difficult to say no 1 2 3 4 5

when people offer

me cigarettes.

k) People like you 1 2 3 4 5

more if you smoke.

l) Smoking helps 1 2 3 4 5

reduce stress.

Go to question 44

44) Below are a few things that people say about themselves. They are true for some people but not for others. **Please say whether each one of these statements is true for you or not.**

**Always true Sometimes true Not true Don’t know**

a) I care about the way I look. 1 2 3 4

b) My parents expect too much 1 2 3 4

of me.

c) I don’t like answering questions 1 2 3 4

in class.

d) It’s hard for me to talk to my 1 2 3 4

friend about my problems.

e) It’s important for me to look 1 2 3 4

grown up.

f) It’s easy to talk to my family 1 2 3 4

about my problems.

g) I make new friends easily. 1 2 3 4

h) I am concerned about what other 1 2 3 4

people think of me.

i) My classmates often tease me. 1 2 3 4

j) I have trouble mixing with people

of my own age. 1 2 3 4

k) I like going to school. 1 2 3 4

l) I usually do well in my lessons. 1 2 3 4

m) I sometimes cut classes. 1 2 3 4

n) I am concerned about my body 1 2 3 4

shape or the way I look.

Go to question 45

45) During the **past four weeks**, how good or bad have you felt about:

(Please tick **ONE BOX ON EVERY LINE**).

**Very good Quite good Not good or bad Quite bad Very bad**

a) yourself? 1 2 3 4 5

b) your school work? 1 2 3 4 5

c) your ability to play sports? 1 2 3 4 5

d) the things you CAN do? 1 2 3 4 5

e) your body and your looks? 1 2 3 4 5

Go to question 46

46) What do you want to do when you leave school? (Please tick **ONE box only**).

Go to college/university 1 Go to question 47

Get a job 2 Go to question 47

Get technical and vocational education training 3 Go to question 47

Get an apprenticeship 4 Go to question 47

Be unemployed 5 Go to question 47

I don’t know 6 Go to question 47

Other (please note what you want to do when you leave school _ _ _ _ _ _ _ _ _ _ _ ) 7

Go to question 47

47) Do you think you will be smoking when you are 16 years old?

(Please tick **ONE box only**).

Yes 1 Go to question 48

No 2 Go to question 48

I don’t know 3 Go to question 48

48) Do you think you will be vaping when you are 16 years old?

(Please tick **ONE box only**).

Yes 1 Go to question 49

No 2 Go to question 49

I don’t know 3 Go to question 49

**[Questions 49-59 below adapted to make relevant for each country to measure socio-economic status]**

49) What is the highest level of schooling completed by your father / male guardian? (Please tick **ONE box only**).

Elementary diploma (or indicated that did not finish elementary) 1 Go to question 50

High diploma 2 Go to question 50

Vocational/technical/2-year degree diploma 3 Go to question 50

College diploma 4 Go to question 50

Master's/doctoral/law /medicine  5 Go to question 50

I don't know 6 Go to question 50

50) What is the highest level of schooling completed by your mother / female guardian? (Please tick **ONE box only**).

Elementary diploma (or indicated that did not finish elementary) 1 Go to question 51

High diploma 2 Go to question 51

Vocational/technical/2-year degree diploma 3 Go to question 51

College diploma 4 Go to question 51

Master's/doctoral/law /medicine  5 Go to question 51

I don't know 6 Go to question 51

51) How much is your weekly allowance? (Please tick **ONE box only**).

I don’t get any pocket money 1 Go to question 52

Less than Php 300 a week 2 Go to question 52

Php 300 or more but less than Php 600 3 Go to question 52

Php 600 or more but less than Php 900 4 Go to question 52

Php 900 or more but less than Php 1200 5 Go to question 52

Php 1200 or more a week 6 Go to question 52

52) Do you have your own cell phone that you do not need to share? (Please tick **ONE box only**).

Yes 1 Go to question 54

No 2 Go to question 53

53) Do you have to share a cell phone? (Please tick **ONE box only**).

Yes 1 Go to question 54

No 2 Go to question 54

54) Do you **usually** pay for the calls/vouchers for your cell phone yourself? (Please tick **ONE box only**).

Yes 1 Go to question 55

No 2 Go to question 55

55) Does your family have a car or van? (Please tick **ONE box only**).

No 1 Go to question 56

Yes, one 2 Go to question 56

Yes, two or more 3 Go to question 56

56) Does your family own a motorcycle? (Please tick **ONE box only**).

No 1 Go to question 57

Yes, one 2 Go to question 57

Yes, two or more 3 Go to question 57

57) Do you have your own bedroom for yourself? (Please tick **ONE box only**).

Yes 1 Go to question 58

No 2 Go to question 58

58) How many computers does your family own (including PCs, Macs, laptops, tablets, iPads, excluding game consoles and phones)? (Please tick **ONE box only**).

None 1 Go to question 59

One 2 Go to question 59

Two 3 Go to question 59

More than two 4 Go to question 59

59) How many times have you and your family travelled out of town for vacation in the past year? (Please tick **ONE box only**).

Not at all 1 Go to question 60

Once 2 Go to question 60

Twice 3 Go to question 60

Three or more times 4 Go to question 60

#### **SECTION FIVE**

#### **This last section is about smoking education & smoking in school. Please answer these questions honestly:**

#### **remember that it is not a test!**

60) In the last school year, have you had any lessons, videos or discussion in class on smoking? (Please tick **ONE box only**).

No 1 Go to question 61

Yes, one 2 Go to question 61

Yes, two or more 3 Go to question 61

61) During school hours, how often do you see or know about students smoking? (Please tick **ONE box only**).

About every day 1 Go to question 62

Sometimes 2 Go to question 62

Never 3 Go to question 62

Don’t know 4 Go to question 62

62) During school hours, how often do you see or know about teachers smoking? (Please tick **ONE box only**).

About every day 1 Go to question 63

Sometimes 2 Go to question 63

Never 3 Go to question 63

Don’t know 4 Go to question 63

63) During school hours, how often do you see or know about other people (e.g. other staff, workmen or visitors) smoking on the school premises? (Please tick **ONE box only**).

About every day 1 Go to question 64

Sometimes 2 Go to question 64

Never 3 Go to question 64

Don’t know 4 Go to question 64

64) Are students allowed to smoke at school? (Please tick **ONE box only**).

No, not at all 1 Go to question 65

Yes, older students are allowed to smoke everywhere 2 Go to question 65

Yes, only older students are allowed to smoke in certain areas 3 Go to question 65

Yes, anywhere on the school premises 4 Go to question 65

Don’t know 5 Go to question 65

65) What action is usually taken if these rules about smoking are broken by students in your school year? (Please tick **AS MANY** boxes as you wish).

Nothing is done about it 1 Go to question 66

Parents are told about it 2 Go to question 66

The student is punished 3 Go to question 66

The student is spoken to about it 4 Go to question 66

Don’t know 5 Go to question 66

66) Are teachers allowed to smoke at school? (Please tick **ONE box only**).

No, not at all 1 Go to question 67

Yes, they are allowed to in certain areas 2 Go to question 67

Yes, anywhere on the school premises 3 Go to question 67

Don’t know 4 Go to question 67

67) How much do you agree with the following statements?

Please tick **ONE box on EACH line**.

**Strongly agree Agree Not sure Disagree Strongly disagree**

a) My teachers are fair 1 2 3 4 5

in dealing with students.

b) There’s at least one 1 2 3 4 5

teacher or other

adut in my school

I can talk to if I have

a problem.

c) In my school,

students’ ideas are 1 2 3 4 5

listened to and valued.

d) In my school, 1 2 3 4 5

teachers and

students really trust

one another.

e) Students at this 1 2 3 4 5

school are

encouraged to take

part in activities,

programs and special

events.

f) I feel close to people

at this school. 1 2 3 4 5

g) I try hard in school. 1 2 3 4 5

Go to question 68

68) The following are a number of statements about your parents/guardians.

Please say how often this happens: (Please tick **ONE box on EACH line**)

**Never Almost never Sometimes Often Almost always or**

**always**

a) If I go out, my 1 2 3 4 5

parents/guardians

tell me what time I

have to be home.

b) My parents/ 1 2 3 4 5

guardians know the

details of what I do in

my free time (e.g.,

where I am going, who

with, and for how long).

c) My parents/ 1 2 3 4 5

guardians check to

see that I have done

my homework.

Go to question 69

69) **Now we would like you to fill out a social network survey. We are collecting this information to learn more about young people's friendships. We will ask for the names of your friends, and then ask a few questions about each person.

All names will be replaced with a code so that they are anonymous. Your friends won't know who you put down.

Please tell us the names of the friends that you currently spend the most time with.

You can list friends from your year-group at your school, friends from outside of school, or even friends who aren't in your grade, but are part of your school.

You don't need to fill in all the spaces.

The names will be kept confidential and we will delete all names to make the data anonymous.**

Friend 1

First name

Second name

Nickname (if they have one)

Friend 2

First name

Second name

Nickname (if they have one)

Friend 3

First name

Second name

Nickname (if they have one)

Friend 4

First name

Second name

Nickname (if they have one)

Friend 5

First name

Second name

Nickname (if they have one)

Friend 6

First name

Second name

Nickname (if they have one)

Friend 7

First name

Second name

Nickname (if they have one)

Friend 8

First name

Second name

Nickname (if they have one)

Friend 9

First name

Second name

Nickname (if they have one)

Friend 10

First name

Second name

Nickname (if they have one)

**We are now going to ask a few questions about each person you named.**

Friend 1

1. Is this person in your grade at your school?

Yes 1 Go to Question 2

No 2 Go to Question 6

If you answered ‘Yes’ to Question 1, please answer questions 2 to 5.

If you answered ‘No’ to Question 1, please answer questions 6 and 7.

2. How close do you feel to them?

Very close 1 Go to Question 3

Close 2 Go to Question 3

Moderately close 3 Go to Question 3

Slightly close 4 Go to Question 3

Not very close 5 Go to Question 3

3. Do you spend time with this person in school?

**YES NO**

1 2

Go to Question 4

4. How often do you spend time with this person after school? Please tick **ONE box only.**

Every day 1 Go to Question 5

Several times a week 2 Go to Question 5

Once a week 3 Go to Question 5

Monthly or less often 4 Go to Question 5

Never 5 Go to Question 5

5. Are you connected to them on social media (e.g., Snap Chat, Tik Tok, Instagram)?

Yes 1 No 2

**If you answered ‘No’ to Question 1 (Is this person in your grade at your school?), please answer questions 6 and 7.**

6. Are they male or female?

Male 1 Female 2 Go to Question 7

7. How old are they?

a. A grade or more younger 1

b. A grade or more older 2

c. The same grade 3

Friend 2

Friend 3

Repeat questions 1-7 up to Friend 10

Friend 10

**Well done, you have finished!**

**Were there any questions you meant to go back and complete? Please check that you have filled in all the questions.**

**Thanks for your help with our research project!**
