# Supplementary material for: A Stop Smoking In Schools Trial in three culturally different middle-income countries (ASSIST global): protocol for a randomised feasibility study
Source: BMJ Open. 2025 Jun 22;15(6):e096963. doi: 10.1136/bmjopen-2024-096963 (PMC12184387; doi:10.1136/bmjopen-2024-096963)
Supplement: online supplemental file 5 [file bmjopen-15-6-s005.docx]

Indicative topic guides for qualitative interviews

Data to be collected from:

1. Peer supporter

2. non-peer supporters

3. Teachers (intervention schools)

4. Trainers

5. Parents

6. Teachers (control schools)

**PEER SUPPORTER**

**Introduction**

- Aims of discussion and ground rules (be respectful; speak one at a time; no right/wrong answers; can choose not to answer/leave at any time; keep what is discussed within group)
- Check: consent completed; permission to record
- Complete brief demographics and any additional paperwork collection

**1. Recruiting peer supporters**

- What did you think of the questionnaires you had to do at the start of the project? [probe: did you like/dislike? Did you understand the questions? **Were there any barriers to honestly answering questions about smoking, for example teachers being present in the room? Did you feel you answered the questions in the questionnaire on smoking honestly**?
- When you were given the nomination questionnaire, what did you think it was about? *[Probe: how easy/hard to think of people; understood questions?]*
- How did you choose who to nominate? *[Probe details]*
- How did you feel about being nominated? *[Probe: why]*
- How did you feel about being asked, once you knew what the role was? *[Probe: why, embarrassed, happy etc; feelings change over time? Reactions of others; other worries]*
- Thinking back to the meeting when you were asked if you wanted to be a peer supporter: was anything said or done at that meeting that really persuaded you to take the role? Was anything said or done that put you off? Was anything confusing or unclear? *[Probe details]*
- What do you think of the group of people who were chosen by this process? *[Probe: any surprises (why)? Suitability to talking about smoking, reaching year group; coverage of year group]*
- What nomination questions would you have asked to get the right PSs for ASSIST?

**2. Using the ASSIST website and or social media platform** (Acceptability, Exposure, Context, Fidelity)

- Was the ASSIST Global website useful?
- Were the suggested messages helpful?
- How did you feel about using a social media platform to post ASSIST messages? *[Probe: any barriers, worries, advantages]*
- What sort of responses did you get to your posts? *[Probe: pos/neg, discussions, questions, or lack of response and how they felt about it, or any incidents of teasing/being silly online]*
- Tell me about the sort of conversations have you had with other people about ASSIST?
- Which way worked better for you: face to face or online? *[Probe: why?]*
- Did anyone tell you anything personal or sensitive during ASSIST? *[No need for details but probe: Did you feel you knew what to do? If you told an adult, was that person helpful? How did YOU feel about this (confident to deal with it; upset etc)]*

**3. ASSIST support and training**

*I would like you to think back to the peer supporter training days, can you tell me a little about what you thought about the training – what comes to mind.*

- What did you think of the trainers? *[Probe: attitude, availability, approachability, clear, knowledge of smoking, reliability, trustworthiness]*
- Did the training give you enough information about smoking to talk to other students about it?
- Did the training give you confidence to talk with other students about smoking?
- What would you change about the training if anything?
- What about the follow-up sessions? *[Probe: Which did you attend? Reasons for (not)attending; (dis)advantages, improvements, problems; probe based on observations]*
- Did you get enough support over the weeks to carry out the peer supporter role? *[Probe: gaps, facilitators’ f2f, online, teachers, trainers]*
- **Did you know that there was an ASSIST Global contact teacher? If yes, did you contact them for anything? Did you find the contact teacher helpful? If not, what could they have done to be more helpful?**
- **Outside of the training and follow up sessions did you contact other peer supporters / discuss ASSIST Global with other peer supporters? If yes, probe specifics.**

**4. Reflections on the peer supporter role** (Acceptability)

- Roughly how many conversations did you have with students in your year group about smoking while you were a peer supporter? Did you find these difficult or easy?
- What, if anything, did you find particularly positive about being a peer supporter? *[Probe specifics]*
- **Did being a peer supporter positively impact you in any way (Probe: e.g. gaining new skills, feeling more confident about expressing your views)?**
- What, if anything, did you find particularly negative about it? *[Probe specifics]*
- Would you volunteer again for a similar role in the future? *[Probe: why? Why not?]*
- Did being a peer supporter affect your relationships with other students in a negative way?
- If a friend was asked to be a ASSIST peer supporter, would you advise them to take the role or not?
- How did the role compare to what you expected it would be like, before you started?
- Would you be interested in continuing the role? *[Probe in what way eg. Same social media platform, new online group with supporters from other schools, other]*
- What do you think would be the challenges to continuing the role? *[probe re requiring trainer support, keeping the messages ‘fresh’, time etc]*
- *Have your views on smoking changed after undertaking this role? [probe: in what ways]*
- Is there anything we haven’t talked about that would have made the peer supporter role easier? Or anything that did make it easy? *[Probe: specifics]*

**5. Response to ASSIST in year group** (Acceptability)

- What, if anything did your (non-peer supporter) friends say to you about the ASSIST or messages you sent? *[Probe: what did they like/dislike, anything funny/boring/irritating/useful etc?]*
- *Did you speak to someone about smoking who hardly ever comes into school? [Probe: when did you have this conversation?, how did they respond?]*
- Can you think of anything that happened in your year because of ASSIST – this could be to do with any changes in friendship groups (people making new friends or falling out or feeling excluded), teasing or bullying, teachers getting involved or asking questions about ASSIST, extra lessons or events, *(probe: positive or negative effects)*

**6. Contextual issues**

- Were there any negative impacts on being a peer supporter because of the COVID situation in your country?
- Philippines: Did the shortening of the school year impact your ability to have conversations with your friends about ASSIST? [probe: did you have conversations with friends after the school year ended? - why/why not]
- Indonesia: Did the timing of the intervention (i.e. during Ramadan) affect whether you were able to have conversations with friends? [probe: were you in contact with as many friends as usual during this time? Did friends reduce smoking during Ramadan?] what might have been different if the intervention had been at another time (not Ramadan)
- Did levels of student attendance affect your ability to have ASSIST discussions? [probes – are there high levels of absenteeism? Were certain students harder to have discussions with? In what ways did this make it more difficult]
- Do any of your friends vape? Did you have discussions about this in relation to ASSIST? [probe – do you think this issue should be covered as part of the ASSIST intervention, if so why, if not why not]
- What do other people outside of your school think about smoking? (i.e. parents, other family and friends). Did you have any discussions with them about ASSIST? [Probes: how did they respond?]

**Close**

- Are there any other issues regarding the ASSIST project that you would like to talk about that we have not had a chance to cover?
- Opportunity for participant to ask questions
- Feedback on interview / THANKS / VOUCHERS

**NON-PEER SUPPORTERS**

**Introduction**

- Aims of discussion and ground rules (be respectful; speak one at a time; no right/wrong answers; can choose not to answer/leave at any time; keep what is discussed within group)
- Check: consent completed; permission to record
- Complete brief demographics and any additional paperwork collection

**1. Recruiting peer supporters** *[have* ***nomination QR*** *to hand]* (Recruitment, Reach, Acceptability)

- What did you think of the questionnaires you had to do at the start of the project? [probe: did you like/dislike? Did you understand the questions?]
- When you were given the nomination questionnaire, what did you think it was about? *[Probe: how easy/hard to think of people; understood questions? Do you see any differences between the questions?]*
- How did you choose who to nominate*? [Probe details]*
- Were you asked to be a peer supporter? *(If yes, probe why they decided not to)*
- What do you think of the group that were chosen to be peer supporters? *[Probe: do they reach across the year group? Were they active? Visible? Responsible? Were the ‘usual suspects’ (the people who always volunteer or get chosen by teachers?]*
- Some say the nomination question is like a popularity contest – what do YOU think?
- Were you disappointed not to be nominated? How did it make you feel?
- What nomination questions would you have asked to get the right peer supporters for ASSIST?

**2. Interaction with peer supporters** (Acceptability, Exposure, Context, Fidelity)

- Were you aware of the ASSIST programme and the peer supporters?
- Did you interact with the peer supporters face to face and discuss smoking related issues? [probe: what did you discuss? What did you think? Did you do anything differently following these conversations?]
- Did you interact with peer supporters online?
- Tell me about how much time you spent looking at messages posted *[Probe change as weeks went on? What kept you engaged? What made you lose interest?]*
- How did you feel about ASSIST messages being shared in this way? *[Probe acceptability of social media platforms; use of social media in school; advantages/disadvantages]*
- Tell me about any face-to-face conversations you had about ASSIST *[Probe what did you discuss and what, if anything did you learn?]*
- If you had both online/phone or face-to-face communication in ASSIST, which did you prefer? *[Probe why?]*
- How do you think your peer supporter managed the role? *[Probe details: active or non-active, approachable, knowledgeable]*
- From your point of view, what could we do to make the peer supporter role better? *[Probe specifics]*
- How could we improve the way ASSIST is run?

**3. Content of ASSIST** (Acceptability)

- Tell me what you thought of the ASSIST topics/messages *[Probe: by topic, likes/dislikes, pos/neg, anything stand out?]*
- What, if any, gaps do you think there were in what was covered in ASSIST? *[Probe details]*
- How relevant/engaging/useful was the information in ASSIST, compared to the smoking / health information you usually get? *[Probe details, specific topics]*
- How could we improve the topics/information covered in ASSIST? *[Probe by topics; relevance]*

**4. Perceptions of data collection**

- What did you think of the ASSIST questionnaire at baseline and follow up? *[Probe: length, acceptability of questions, concerns about privacy, comprehension etc]*
- Did you feel you answered the questions on smoking honestly? Did you find answering questions about smoking with teachers present difficult?

**5. ASSIST on the whole**

- Can you tell me about anything particularly good that you think came out of ASSIST? *[Probe specifics; discussion, group interaction, changes in attitudes]*
- Can you tell me about anything particularly negative that came out of ASSIST? *[Probe difficulties, challenges, disagreements, negative behaviour]*
- If a friend at another school was asked to take part in ASSIST, what would you tell them to do? *[Probe reasons]*
- Would you have liked to have been a peer supporter? *[Probe reasons]*
- Would you like to stay involved in ASSIST in any way? *[Probe in what way eg. continuing online group]*

**Close**

- Is there anything else to do with ASSIST that you’d like to talk about? That we have not had a chance to cover
- Opportunity for participant to ask questions / feedback
- THANKS / VOUCHERS

**TEACHERS (intervention schools)**

**Introduction**

- Aims of discussion and ground rules (no right/wrong answers; can choose not to answer/leave at any time)
- Check: consent completed; permission to record
- Complete brief demographics and any additional paperwork collection, eg. Job title, key responsibilities, any involvement with ASSIST

**1. Initial impressions of ASSIST [as applicable]** (Acceptability, Context, Fidelity)

- What were your first impressions of ASSIST when you heard about it? *[Probe details]*
- What were your impressions of the ASSIST questionnaire sessions? *[Probe organisation of session, content of QR, how involved, Qs from students]*

**2. Peer supporter nomination** (Acceptability, Reach, Context)

- What were your impressions of the peer nomination process? *[Probe specifics: get the ‘right’ students; any groups excluded? How process is perceived by staff/students?]*
- What are your overall impressions of the group selected by the nomination process? *[Probe any surprises, coverage of year group, gaps]*
- Were you aware of any nominated students who chose not to take up the role? *[Probe reasons]*

**3. Training and follow-up [as applicable]** (Fidelity, Acceptability, Context)

- What was your impression of:
  - Peer supporter Training? *[Probe: content, format, relevance to S4s, student interaction, barriers/facilitators]*
  - Follow-up sessions? *[Probe: content, format, interaction, barriers/facilitators]*
  - Trainers *[Probe interaction with students, ability to facilitate the session/keep on task]*
- From your point of view, were peer supporters provided with sufficient support to do the role? *[Probe gaps]*

**4. Content of ASSIST** (Acceptability)

- Tell me what you thought of the smoking / health information provided in ASSIST *[Probe: by on familiarity, anything stand out?]*
- What, if any, gaps do you think there were in what was covered in ASSIST? *[Probe details]*
- How would you compare the information provided in ASSIST to that normally taught at your school? *[Probe details, specific topics; whether complemented, overlapped]*
- How could we improve the information covered in ASSIST? *[Probe by topics; relevance]*

**5. Young person’s response to the intervention**

- How do you think the year group responded to ASSIST on the whole?
- Were you aware of anyone being excluded from ASSIST? *[Probe: reasons, barriers, facilitators]*
- Were you aware of any specific issues encountered by peer supporters? *[Probe details e.g. bullying, misbehaviour on line, younger years getting access to website; parental complaints or concerns]*
- Can you tell me about anything particularly good that you think came out of ASSIST? *[Probe specifics; discussion, group interaction, changes in attitudes]*
- Can you tell me about anything particularly negative that came out of ASSIST? *[Probe difficulties, challenges, disagreements, negative behaviour]*
- As far as you are aware, did any untoward incidents or sensitive disclosures occur as a result of ASSIST? *[No need for names, but details and who/how managed]*
- Was there any other disruption or cost to the school, not already covered? *[Probe specifics, solutions]*

**5. Implementing ASSIST in schools** **[as applicable]** (Acceptability, Context)

- What other programmes or research were the school involved in, other than ASSIST?
- How much awareness would you say there was across the school about ASSIST? *[Probe year group, beyond year group, staff]*
- From your perspective, how well did ASSIST integrate into day-to-day school life? *[Probe: disruption; changes in social dynamics; chat in staff room; taking up staff time, unexpected issues]*
- What are your views on the acceptability of what the school was asked to do? [*Probe time commitment, resources; data collection sessions, training/follow-up]*
- Is there anything that you think could be a barrier to schools participating in ASSIST?
- Or anything that would/does facilitate schools’ participation?
- What are your views on the level of communication between the ASSIST team and the school? *[Probe: researchers and school; ASSIST trainers and school]*
- How did the reality of ASSIST compare to your expectations, prior to it starting?
- Would you see value in maintaining the school’s involvement in ASSIST? *[Probe current student group, future years; online, discussion groups, how best to embed in school]*
- Would you be interested in using this group of peer supporters in other ways? *[e.g. to teach pupils about other health behaviour topics, probe on other ways that the group could work and their challenges]*
- Were there any changes to health/smoking education in school over the time that ASSIST was running or any other initiatives that might have affected how ASSIST went? *[Prompt specifics, potential implication for ASSIST]*
- Did being part of ASSIST lead to your school doing anything differently in terms of health/smoking education?
- As far as you are aware, did any untoward incidents or sensitive disclosures occur as a result of ASSIST? *[No need for names, but details and who/how managed]*

**6. Contextual issues**

- Was there any negative impacts on how the ASSIST Global intervention was delivered/worked because of the COVID situation in your country?
- Philippines: Do you think the shortening of the school year impacted the peer supporters delivery of the intervention? [probe: in what ways]
- Indonesia: Do you think the timing of the intervention (i.e. during Ramadan) affected the delivery of the intervention? [probe: in what ways?]
- Do you think there are any other issues which may impact how ASSIST was delivered and received in your school? [Probe: availability of students, vaping, other student/faculty/parental attitudes and behaviours towards smoking]

**Close**

- Are there any other issues regarding ASSIST that you wish to raise?
- Opportunity for participant to ask questions / feedback on interview
- Thanks and wrap up

**ASSIST Trainers**

**Introduction**

- Aims of discussion and ground rules (no right/wrong answers; can choose not to answer/leave at any time)
- Check: consent completed; permission to record
- Complete brief demographics and any additional paperwork collection, eg. Job title, key responsibilities, extent of involvement with ASSIST eg, number of sessions led

**1. ASSIST PEER SUPPORTERS**

- Thinking back to the recruitment meetings, was there anything that helped to make these run particularly well? *[Probe contextual factors, room, staff present, timing, characteristics of group]*
- Any barriers to their running well? *[Probe as above]*
- What was your overall impression of the peer supporter groups *[Probe by each school worked with; specific characteristics, dynamics, mix, friend groups, willingness to engage, maturity]*

**2. ASSIST Training**

- Thinking of a training session that went really well, what helped it to go well? *[probe: school support, group dynamics, venue etc]*
- Thinking of training sessions that did not go well, why was that? *[probe: school support, group dynamics, venue etc]*
- Thinking back across all the training sessions you were involved in, which components do you feel:
  - Worked best / were most acceptable to Peer Supporters? *[Probe why]*
  - Worked least well / were least acceptable to the Peer Supporters? *[Probe why]*
- To what extent do you think the training activities adequately prepared Peer Supporters for the role? *[Probe skills, knowledge, confidence, gaps]*
- Tell me about any adaptations you had to make to the training content/format *[Probe which school, why, outcome; use to build on any adaptations noted in observations]*
- How could we improve the Peer Supporters training? *[Probe specifics, reasons]*
  - Are there any other issues that you want to note regarding the training? *[Probe for particular challenges content, delivery]*

**3. Follow-up sessions and ongoing support** (Acceptability, Context, Fidelity)

- To what extent do you feel the follow-up sessions helped Peer Supporters to deliver:
  - *the online component of ASSIST*
  - *face-to-face conversations about ASSIST [Probe for each session aim as per Trainer Manual]*
- Thinking back across all the follow-ups you were involved in, which components do you feel:
  - Worked best / were most acceptable to the Peer Supporters? *[Probe why]*
  - Worked least well / were least acceptable to the Peer Supporters? *[Probe why]*
- To what extent do you think the follow-up sessions adequately supported Peer Supporters? *[Probe re skills, knowledge, confidence]*
- Tell me your views on the practical arrangements for the follow-up sessions *[Probe re. school management/accommodation of needs; teacher involvement]*
- Tell me about any common questions Peer Supporters had about ASSIST *[Probe who, details, response]*
- Are there any other issues that you would like to note regarding the follow-up sessions? *[Probe content, delivery; particular challenges]*
- How could we improve the follow-up sessions? *[Probe: for the trainers, for the PSs]*
- Tell me about any support you had to provide Peer Supporters via the support forum? *[Probe specific contacts, questions, requests, interventions]*
- To what extent do you feel your membership of this group was an useful way to support the peer supporters? *[Probe why, issues, alternatives]*
- Tell me about any issues that occurred in the course of moderating the group *[Probe response, resolution]*
- Based on what you saw when moderating group posts, tell me what you can about the extent to which peer supporters engaged with ASSIST *[Probe posts, adaptations etc]*
- Tell me about any questions you were sent directly on the forum *[Probe nature of question; characteristics of sender, response]*
- Based on your experience, how might we improve the ASSIST forum?
- How time-consuming for you was the forum support component of ASSIST *[Probe per day/week; more/less than expected; acceptability]*
- How could we improve the different components of ASSIST *[Probe eg. supporting the PSs, communication]*
- As far as you are aware, did any untoward incidents or sensitive disclosures occur as a result of ASSIST? *[No need for names, but details and who/how managed]*

**4. ASSIST website**

- **Did you look at the ASSIST website?**
- **What did you think of it, was it useful?**
- **Did you encourage the peer supporters to use the website?**
- **Did you use the website during the training sessions, e.g. to demonstrate it to the peer supporters?**
- **Did you show the students where the shareable content was on the website and encourage them to use these prompts when talking about not smoking with their friends?**
- **Do you think the peer supporters used the website? If not, why not, if so ,why so?**
- **Based on your experience, how might we improve the ASSIST website?**

**5. Working with schools** (Acceptability, Context)

- In which schools did ASSIST go particularly well and why do you think that was? *[Probe: support from contact teacher, school buy in, organisation/administration, dynamics between peer supporters]*
- In which schools did ASSIST go not so well and why do you think that was? *[Probe: support from contact teacher, school buy in, organisation/administration, dynamics between peer supporters]*

**6. Acceptability of Trainer role** (Acceptability, Context, Fidelity)

- Did you feel sufficiently prepared for your role in ASSIST? *[Probe recruitment, training, suggestions for improvement]*
- To what extent did you feel equipped to manage any questions put to you by peer supporters that were:
  - Smoking / health-related *[Probe: in training, follow up, online; specifics]*
  - ASSIST skills-related? *[Probe: in training, follow up, online specifics?]*
- Where there any activities that you felt less comfortable in delivering? *[Probe training, follow-up; least/most acceptable components; specifics, why; how could this be improved on,]*
- Where there any follow-up sessions that you were less comfortable in delivering? *[Probe training, follow-up; least/most acceptable components; specifics, why; how could this be improved on]*
- Did the ASSIST team provide enough support for your role in supporting PSs online? *[Probe gaps, suggestions for improvement]*
- Were any of the aspects of the trainer role not acceptable to you? *[Probe why?]*

**7. COVID issues**

- Was there any negative impacts on how the ASSIST Global intervention was delivered/worked because of the COVID situation in your country?
- Philippines: Do you think the shortening of the school year impacted the peer supporters delivery of the intervention? [probe: in what ways]
- Indonesia: Do you think the timing of the intervention (i.e. during Ramadan) affected the delivery of the intervention? [probe: in what ways?]
- Do you think there are any other issues which may impact how ASSIST was delivered and received? [Probe: availability of students, vaping, other student/faculty/parental attitudes and behaviours towards smoking]

**Close**

- Are there any other issues regarding ASSIST that you wish to raise?
- Opportunity for participant to ask questions / feedback on interview
- Thanks and wrap up

**Parents**

**Introduction**

- Aims of discussion and ground rules (no right/wrong answers; can choose not to answer/leave at any time)
- Check: consent completed; permission to record
- Complete brief demographics and any additional paperwork collection

**1. Initial impressions of ASSIST [as applicable]** (Acceptability, Context, Fidelity)

- What were your first impressions of ASSIST when you heard about it? *[Probe details, what did your child tell you about this?]*

**2. Peer supporter nomination** (Acceptability, Reach, Context)

- Were you aware of how peer supporters were chosen? Did your child mention this process?
- What were your impressions of the peer nomination process? *[Probe specifics: get the ‘right’ students; any groups excluded? How process is perceived by staff/students?]*
- Were you aware your child was nominated by other students to take up the role? *[Probe how did you feel, child feel?]*

**3. Training and follow-up** (might not be applicable) (Fidelity, Acceptability, Context)

- If your child took on the peer support role, what was your impression of:
  - Peer supporter Training? *[Probe: content, format, relevance, student interaction, barriers/facilitators]*
  - Follow-up sessions? *[Probe: content, format, interaction, barriers/facilitators]*
  - Trainers *[Probe interaction with students, ability to facilitate the session/keep on task]*
- From your point of view, were peer supporters provided with sufficient support to do the role? *[Probe gaps]*

**4. Content of ASSIST** (if aware) (Acceptability)

- Tell me what you thought of the smoking / health information provided in ASSIST *[Probe: by on familiarity, anything stand out?]*
- What, if any, gaps do you think there were in what was covered in ASSIST? *[Probe details]*
- How could we improve the information covered in ASSIST? *[Probe by topics; relevance]*

**5. Young person’s response to the intervention**

- How do you think your child responded to ASSIST on the whole?
- Were you aware of any specific issues encountered by your child or their friends? *[Probe details e.g. misbehaviour on line, bullying, younger years getting access to website; parental complaints or concerns]*
- Did you child talk to you about ASSIST at home *[Probe specifics; changes in attitudes, changes at home]*
- Can you tell me about anything particularly good that you think came out of ASSIST; whether your child was a peer supporter or a non-peer supporter? *[Probe specifics; discussion, changes in attitudes, changes at home]*
- Can you tell me about anything particularly negative that came out of ASSIST; whether your child was a peer supporter or a non-peer supporter? *[Probe difficulties, challenges, disagreements, negative behaviour]*
- As far as you are aware, did any untoward incidents or sensitive disclosures occur as a result of ASSIST? *[No need for names, but details and who/how managed]*

**5. Implementing ASSIST in schools** **[as applicable]** (Acceptability, Context)

- How much awareness would you say there was across the school about ASSIST? *[Probe year group, beyond year group, staff, did you hear other children or parents talking about it]*
- From your perspective, how well did ASSIST integrate into day-to-day school life? *[Probe: disruption; changes in social dynamics; chat in staff room; taking up staff time, unexpected issues]*
- What are your views on the acceptability of what the school was asked to do? [*Probe time commitment, resources; data collection sessions, training/follow-up]*
- Is there anything that you think could be a barrier to schools participating in ASSIST?
- Or anything that would/does facilitate schools’ participation?

**Close**

- Are there any other issues regarding ASSIST that you wish to raise?
- Opportunity for participant to ask questions / feedback on interview
- Thanks and wrap up

**TEACHERS (Control schools)**

**Introduction**

- Aims of discussion and ground rules (no right/wrong answers; can choose not to answer/leave at any time)
- Check: consent completed; permission to record
- Complete brief demographics and any additional paperwork collection, eg. Job title, key responsibilities, any involvement with ASSIST

**1. Initial impressions of ASSIST [as applicable]** (Acceptability, Context, Fidelity)

- What were your first impressions of ASSIST when you heard about it? *[Probe details]*
- What were your impressions of the ASSIST questionnaire sessions? *[Probe organisation of session, content of QR, how involved, Qs from students]*

**2. Peer supporter nomination** (Acceptability, Reach, Context)

- What were your impressions of the peer nomination process? *[Probe specifics: get the ‘right’ students; any groups excluded? How was the process perceived by staff/students?]*

**3. School being allocated to the control condition** (Acceptability, Context)

- How did you feel when the school was randomised as a control school? [Probe – e.g. disappointed – why?]
- In what ways do you think the ASSIST intervention would have had an impact on the school? [Probe details, what were your expectations of the ASSIST trial?]
- What is “normal” practice in your school regarding smoking prevention or education initiatives? [probe details]

**5. Young person’s smoking behaviours during the trial period (Context)**

- Have young people's smoking behaviours changed since the ASSIST questionnaires were performed? [probe – has smoking rates increased/decreased/stayed the same – if there is a change, what do you think caused this?]
- Do you feel students' attitudes towards smoking has changed in any way? [probe – in what ways? What influenced this?]
- Has the school delivered any additional education or prevention initiatives since the ASSIST questionnaires were performed? (probes – have any smoking events occurred, if yes – do these usually occur, did they influence student smoking behaviour?)

**6. Contextual issues**

- Do you think the ASSIST intervention has influenced smoking behaviour and attitudes in your school in any unexpected ways even though you were in the control condition? (e.g. knowing students who are at an intervention school, the baseline questionnaires)
- Do you think there are any other issues which may impact smoking behaviours in your school? [Probe: availability of students, vaping, religious festivals and holidays, other student/faculty/parental attitudes and behaviours towards smoking]

**Close**

- Are there any other issues regarding ASSIST that you wish to raise?
- Opportunity for participant to ask questions / feedback on interview
- Thanks and wrap up
