# Supplementary material for: A Stop Smoking In Schools Trial in three culturally different middle-income countries (ASSIST global): protocol for a randomised feasibility study
Source: BMJ Open. 2025 Jun 22;15(6):e096963. doi: 10.1136/bmjopen-2024-096963 (PMC12184387; doi:10.1136/bmjopen-2024-096963)
Supplement: online supplemental table 1 [file bmjopen-15-6-s007.docx]

Table 1 Country-level Progression criteria from feasibility trial to full randomised controlled trial

|  | Criteria | INDICATOR*  GREEN=Very strong indication to proceed  AMBER=Medium indication to proceed. Discuss with TSC and proceed with identified plan to improve performance on indicator in Phase III trial  RED=Indication of doubt as to whether to proceed. Discuss with TSC, and only proceed if other indicators are amber/green and there is a clear mitigating strategy  (NB these are listed in order of green, amber, red below) |
| --- | --- | --- |
|  | 1. INTERVENTION ACCEPTABILITY AND FIDELITY | |
|  | INTERVENTION SCHOOLS ONLY (N=6) | |
| 1 | Was it feasible to recruit and train peer supporters? | In at least two thirds of recruited intervention schools, 70% or more nominated students are recruited and complete the training. |
|  |  | In at least two thirds of recruited intervention schools, 60-69% of nominated students are recruited and complete the training **OR** the social network analysis shows that the peer supporters reached most of the year group. |
|  |  | Amber criteria not attained. |
| 2 | Were peer supporters able to carry out the role? | In at least four intervention schools 60% or more peer supporters who complete the training, send three or more messages or have three or more conversations, and attend two or more follow-up meetings |
|  |  | In at least four intervention schools 50-59% of those who complete the training, send three or more messages/have three or more conversations, and attend two or more follow-up meetings |
|  |  | Amber criteria not attained. |
| 3 | Did the wider target group find the ASSIST intervention useful? | 50% or more of those who reported smoking or experimenting with smoking, and talked to a Peer Supporter, indicated that they found the intervention useful. |
|  |  | 30-49% of those who reported smoking or experimenting with smoking, and talked to a Peer Supporter, indicated that they found the intervention useful. |
|  |  | Amber criteria not attained. |
| 4 | Was the ASSIST Global intervention acceptable to parents/carers of peer supporters? | 85% or more peer supporters report that their parents were happy about them being a peer supporter. |
|  |  | 70-84% of peer supporters report that their parents were happy about them being a peer supporter. |
|  |  | Amber criteria not attained. |
| 5 | Was the ASSIST Global intervention acceptable to participating schools? | No major acceptability issues raised by participating schools. |
|  |  | Up to 2 major acceptability issues that **can** be mitigated (i.e. none that cannot be mitigated) raised by participating schools. |
|  |  | At least one major acceptability issue raised by schools for which there is no possible mitigating strategy. |
|  | B. SCHOOL AND PARTICIPANT RECRUITMENT AND RETENTION | |
|  | INTERVENTION AND CONTROL SCHOOLS (N=10) | |
| 6 | Did we manage to recruit the required number of schools? | 10 schools recruited |
|  |  | 6-8 schools recruited |
|  |  | Amber criteria not attained. |
| 7 | Was the ASSIST Global study methodology acceptable to participating schools? | No major acceptability issues raised by participating schools. |
|  |  | Up to 2 major acceptability issues that **can** be mitigated (i.e. none that cannot be mitigated) raised by participating schools. |
|  |  | At least one major acceptability issue raised by schools for which there is no possible mitigating strategy. |
| 8 | Was it possible to retain schools? | All 10 schools retained till follow-up. |
|  |  | 8-9 schools retained at follow-up. |
|  |  | Amber criteria not attained. |
|  | C. PRIMARY OUTCOME MEASUREMENT | |
| 9 | Was opt out rate acceptable (N students completing questionnaires)? | Parents opted out 10% or fewer students. |
|  |  | Parents opted out between 11-20% of students. |
|  |  | Amber criteria not attained. |
| 10 | Were the questionnaires acceptable and feasible to students? | Average student response rate across intervention and control schools of 70% or more on the primary outcome. |
|  |  | Average student response rate across intervention and control schools of 60-69% on the primary outcome. |
|  |  | Amber criteria not attained. |
| 11 | Is the primary outcome likely to be accurately measuring smoking? | Using the different sources of evidence in the next column, the study team and TSC will make a judgement on this criterion. More detail on this will be described in the Statistical Analysis Plan (no cut-offs will be used). |
