# Supplementary material for: A Stop Smoking In Schools Trial in three culturally different middle-income countries (ASSIST global): protocol for a randomised feasibility study
Source: BMJ Open. 2025 Jun 22;15(6):e096963. doi: 10.1136/bmjopen-2024-096963 (PMC12184387; doi:10.1136/bmjopen-2024-096963)
Supplement: Supplementary file 8 [file bmjopen-2024-096963.reviewer_comments.pdf]

PEER REVIEW HISTORY

BMJ Open publishes all reviews undertaken for accepted manuscripts. Reviewers are asked to complete a checklist review form and are provided with free text boxes to elaborate on their assessment. These free text comments are reproduced below.

ARTICLE DETAILS

Title (Provisional)

A Stop Smoking In Schools Trial in three culturally different middle-income countries (ASSIST Global): protocol for a randomised feasibility study.

Authors

Soe, Yin Nwe; Thomson, Meigan; Reid, Kate; Moore, Laurence; Bintoro, Bagas Suryo; Dong, Bin; Good, Sally; Hu, Peijin; Long, Emily; Mateo, Nino Jose; McMeekin, Nicola; Padmawati, Retna Siwi; Prabandari, Yayi Suryo; Purvis, Anthony; Salanga, Maria Guadalupe; Semple, Sean; Llorin, Charisse Tan; Wang, Jing-Yi; Simpson, Sharon Anne Anne

---

VERSION 1 - REVIEW

**Reviewer**	**1**
**Name**	**Sanchez-Franco, Sharon**
**Affiliation**	**Universidad de los Andes, School of Medicine**
**Date**	**31-Dec-2024**
**COI**	**None**

The protocol describes a school-level randomized trial to assess the feasibility of a smoking intervention that has been designed and tested in the UK in two low- and middle-income Asian countries. The paper is clear and complete. The study is very relevant for similar settings, and it will be able to contribute to the global literature on adolescent tobacco control, health promotion, and implementation science. In coherence with the study goals, I strongly suggest giving more details regarding the context of the study settings, the intervention, and the adaptation of the interventions.

As this study has a wide range of cross-cultural characteristics, it will necessarily specify how this will be managed. In the future, I hope to know the findings of this study. Following, I detail comments and recommendations.

- References are very unusual in abstracts. Please ensure the abstract is a summary of the paper using the author’s words.

- P2, line 8. (Same in P5, line 9.) “Around 80% of the world's smokers live in lower-middle income countries and smoking rates in China, Philippines, and Indonesia are very high (1, 2).” Data from references published in 2012 are outdated. Please, update this data using the most recent global reports of tobacco, for example, the WHO report on the global tobacco epidemic 2023.

- P2, line 47. “Statistical analyses will focus on feasibility criteria and qualitative data will be analyzed using a framework approach” Please, clarify the purpose of the qualitative component in order to give a balance between both methods. What is the study object in qualitative analysis?

- P5, lines 10-24. As tobacco epidemic monitoring varies across countries, it is relevant to clarify the date of surveys for each. I can see that some countries like Indonesia have available data for 2023, but other countries haven’t. So, please, update the data when it is possible and clarify the availability of data for each country.

- As this study is focused on a feasibility study of an intervention in different cultures, I strongly suggest including subtitles to develop both of the following issues: 1) a short description of the ASSIST intervention, and 2) study settings. The current description of ASSIST avoids understanding why this intervention could contribute to tobacco control in contrast with traditional health education programs. In addition, mixing the context data gives a false apparency that contexts and culture are very similar in the three countries. However, the differences across countries will be very relevant for the adaptation and implementation of the intervention because this will interact with the context. It is very important to understand the intervention that is a study object in this research as well as the description of each context including, the tobacco epidemic, regulations, and education system.

- Governmental, institutional, and community approvals are part of the feasibility and implementation of health interventions. I suggest keeping the documentation of the attempt of the study in China in order to inform how stakeholders and regulation at the organizational level could affect health education interventions and their evidence-based evaluation. Literature in this regard is very limited, and this kind of situation is very common in LMICs.

- P7, lines 35-43. Please specify the sampling methods and expected sample for the qualitative component.

- P8, Fig 1. Please, clarify what is the denominator of the 18%. Does it correspond to the whole school, a year group according to a tailored age, or a subgroup of the influential students?

- P9, line 41. “No single-sex schools (due to the low rates of girls smoking in some countries and also the important influence of the opposite sex on smoking behavior”. Please, clarify

the sex-related exclusion criteria for schools. Does this mean that just male schools will be included? If yes, please justify it in the introduction and adjust the aims of the study.

- P11, Fig 2. Please, name both columns and define the color convention (blue and red letters)

- P13, line 56. Please describe the usual health-education-program in each country. Is there any theory behind them? What is the expected mechanism of action? What are the expected outcomes? What are the main characteristics of their implementation? How long have they been implemented? Is there any evidence-based evaluation of these programs?

- P21, lines 37-55. It is not clear what the purpose of the qualitative component is, nor how it is related to the quantitative component. Please, clarify how both methods interact with each other. If the qualitative component does not prevail over the quantitative component, please relocate the description of the qualitative component (or check the heading style) and describe a methodological strategy for the integration/interaction of both components.

- P25, lines 29-33. Please describe how researchers will handle the language differences with original data during the qualitative analysis, particularly in the "double coding by the UK team".

- As this study applies an intervention designed in the UK to contrasting settings in at least two LMIC Asian countries, the protocol should comprehensively describe the program's cultural adaptation prior to the main study. A few lines at the end of the document briefly mention adaptation of study materials. I strongly suggest detailing how the program will be adapted for implementation in both countries (Indonesia and the Philippines). How will modifications be decided and executed? Will program fidelity be assessed? If so, how?

- Similarly, the protocol does not address how cultural and language differences will be managed during program implementation, data collection, and analysis. The study settings and participants have unique cultural and linguistic characteristics that appear to be overlooked. While it is likely the researchers are considering these differences, it is essential to describe their plans to ensure replicability.

- Evidence-based experiences of ASSIST implementation in LMICs (e.g., in Latin America) are available in the literature. Consulting or referencing these experiences could provide valuable insights for this study.

VERSION 1 - AUTHOR RESPONSE

8.

"References are very unusual in abstracts. Please ensure the abstract is a summary of the paper using the author's words."

The references have been deleted. We are unsure what is meant by ‘using the author words’ as we have already done this when writing the abstract.

9.

“P2, line 8. (Same in P5, line 9.) “Around 80% of the world's smokers live in lower-middle income countries and smoking rates in China, Philippines, and Indonesia are very high (1, 2).” Data from references published in 2012 are outdated. Please, update this data using the most recent global reports of tobacco, for example, the WHO report on the global tobacco epidemic 2023.”

We have removed the 2012 reference and added update references. The WHO report on tobacco 2023 has also been added.

10.

“P2, line 47. “Statistical analyses will focus on feasibility criteria and qualitative data will be analyzed using a framework approach” Please, clarify the purpose of the qualitative component in order to give a balance between both methods. What is the study object in qualitative analysis?”

We feel that the purpose is already stated where we say – “A mixed methods process evaluation will assess acceptability, feasibility, and fidelity of intervention delivery, exposure to and reach of the intervention.”

The process evaluation is primarily qualitative and we feel the objectives are covered under ‘mixed methods’ and we don’t think we need to further clarify that mixed methods are both qualitative and quantitative.

11.

“P5, lines 10-24. As tobacco epidemic monitoring varies across countries, it is relevant to clarify the date of surveys for each. I can see that some countries like Indonesia have available data for 2023, but other countries haven’t. So, please, update the data when it is possible and clarify the availability of data for each country.”

We have now added the most up to date data we are aware of.

12.

“As this study is focused on a feasibility study of an intervention in different cultures, I strongly suggest including subtitles to develop both of the following issues: 1) a short description of the ASSIST intervention, and 2) study settings.”

Regarding the ASSIST intervention – we have now added a brief description on p5.

Further detail on the intervention is available on p11 under ‘Intervention group”.

We have added some context on the countries taking part to the introduction and believe that there is enough information in the introduction to describe the study settings and why they have been

chosen (high rates of smoking, no smoking cessation programmes for adolescents). More detail on the settings is in the section further down on “Trial setting”.

13.

“The current description of ASSIST avoids understanding why this intervention could contribute to tobacco control in contrast with traditional health education programs.”

On pages 4 and 5 we have made clear why we think this intervention could contribute to tobacco control in contrast to traditional health education – i.e.,

“There are few or no smoking cessation programmes for adolescents in these countries (21). However, while tobacco control policies are crucial, public health practitioners recognise the need for interventions at multiple socio-ecological levels (22).”

“While both countries have smoke-free school rules, there are variations in enforcement and in the provision of school-based smoking prevention programmes. When implemented, these programmes are often infrequent, inconsistent and, adult-led which are less effective than those led by peers (21).”

Also: “*ASSIST (A Stop Smoking In Schools Trial) is a ‘peer-led’, school-based smoking prevention intervention, developed in the UK and shown to be effective in preventing adolescent smoking uptake (4) when the UK had insufficient tobacco control measures, and high rates of smoking, which were rising in females (26) - similar to the current situation in the study sites.”*

14.

“In addition, mixing the context data gives a false apparency that contexts and culture are very similar in the three countries. However, the differences across countries will be very relevant for the adaptation and implementation of the intervention because this will interact with the context. It is very important to understand the intervention that is a study object in this research as well as the description of each context including, the tobacco epidemic, regulations, and education system.”

We agree that the countries are all very different in terms of context and culture, which was one reason for choosing these countries. The differences will be explored - as stated in the process evaluation - when looking at contextual factors operating at different levels.

We believe we have described the tobacco epidemic in each country already in the introduction, as well as regulations and that adding a separate section on study settings would interrupt the flow of the narrative. However, we have added some additional details on country contexts on page 4 which we hope addresses this point.

15.

“Governmental, institutional, and community approvals are part of the feasibility and implementation of health interventions. I suggest keeping the documentation of the attempt of the study in China in order to inform how stakeholders and regulation at the organizational level could affect health education interventions and their evidence-based evaluation. Literature in this regard is very limited, and this kind of situation is very common in LMICs.”

We agree with this comment and have kept this information on page 5.

16.

"P7, lines 35-43. Please specify the sampling methods and expected sample for the qualitative component."

This information can be found under "Qualitative data" on pages 21-22.

17.

"P8, Fig 1. Please, clarify what is the denominator of the 18%. Does it correspond to the whole school, a year group according to a tailored age, or a subgroup of the influential students?"

We have added text in Fig. 1.

The denominator for the 18% rate is the number of pupils nominated to be peer supporters by their year group. The equation for this is: 18% = (# of students invited to recruitment meeting/# of students nominated to be a peer supporter).

18.

"P9, line 41. "No single-sex schools (due to the low rates of girls smoking in some countries and also the important influence of the opposite sex on smoking behavior". Please, clarify the sex-related exclusion criteria for schools. Does this mean that just male schools will be included? If yes, please justify it in the introduction and adjust the aims of the study."

On page 9 we describe the study inclusion and exclusion criteria. We are excluding single-sex schools.

We state: "*No single-sex schools are to be included in the study due to a number of considerations" - e.g., female-only schools are excluded due to the low rates of girls smoking in some countries; both female- and male-only schools are excluded as it is important for the study to consider the influence of the opposite sex on smoking behaviour."*

19.

"P11, Fig 2. Please, name both columns and define the color convention (blue and red letters)"

This has been completed and a new supplementary file containing updated Fig 2 has been uploaded.

20.

"P13, line 56. Please describe the usual health-education-program in each country. Is there any theory behind them? What is the expected mechanism of action? What are the expected outcomes? What are the main characteristics of their implementation? How long have they been implemented? Is there any evidence-based evaluation of these programs?"

Thank you for this suggestion, we have added a description of these under “control group” p13. We weren’t able to get some of the details requested above relating to theory and mechanisms or length of delivery or evaluation for these interventions. We will be exploring this further as part of the process evaluation and our planned policymaker interviews and will endeavour to get more information on these and report in due course.

21.

“P21, lines 37-55. It is not clear what the purpose of the qualitative component is, nor how it is related to the quantitative component. Please, clarify how both methods interact with each other. If the qualitative component does not prevail over the quantitative component, please relocate the description of the qualitative component (or check the heading style) and describe a methodological strategy for the integration/interaction of both components.”

The purpose of the qualitative component is described on p21 under ‘Qualitative data’.

We thank the reviewer for the opportunity to clarify the mixed methods approach. We have added a section on this on page 25.

22.

“P25, lines 29-33. Please describe how researchers will handle the language differences with original data during the qualitative analysis, particularly in the “double coding by the UK team”.”

The transcripts are translated and checked by local study team before being transferred to the UK team for double coding. We have edited the text to highlight this on page 25.

23.

“As this study applies an intervention designed in the UK to contrasting settings in at least two LMIC Asian countries, the protocol should comprehensively describe the program’s cultural adaptation prior to the main study. A few lines at the end of the document briefly mention adaptation of study materials. I strongly suggest detailing how the program will be adapted for implementation in both countries (Indonesia and the Philippines). How will modifications be decided and executed?”

We thank the reviewer for this comment, we are aware of the need for adaptation of the intervention and this is included as a key objective of the study: “What, if any, *adaptations* to ASSIST as previously delivered in the UK, are required for its delivery in these countries given the different cultural, political, social, and educational systems?”

As this is the protocol paper describing what is planned with no ‘results’, these pre-trial adaptations will be reported in the main trial paper. The process of deciding these involved extensive consultation with partners and our advisory groups. The trial team made final decisions on these as to what was feasible to implement and also what could be implemented while retaining the key mechanisms in the programme theory. Changes include adaptations to the training activities, the venue and timing of the training, the instructions to peer supporters to accommodate local language and culture, and related materials (e.g., liquorice being changed to menthol in Indonesia and angelica root in China). These were suggested by our country partners our Young People’s Advisory

Group and Stakeholder Advisory Group, - see pages 25 and 26. We have added something on the process on page 25/26 which we hope addresses this comment.

24.

“Will program fidelity be assessed? If so, how?”

This is described on p21 and p 24 under ‘Observations’. The sessions are being filmed and will be rated by an expert using a checklist, to provide an assessment of intervention fidelity.

25.

“Similarly, the protocol does not address how cultural and language differences will be managed during program implementation, data collection, and analysis. The study settings and participants have unique cultural and linguistic characteristics that appear to be overlooked. While it is likely the researchers are considering these differences, it is essential to describe their plans to ensure replicability.”

Sensitivity to recognise and if necessary adapt to local language and cultural differences will be considered throughout the trial and program implementation and form a key objective of the study. Our advisory groups and country partners will advise on all aspects related to language and culture. These pre-trial adaptations will be reported in the main trial paper. Also see above reply to point 23.

26.

“Evidence-based experiences of ASSIST implementation in LMICs (e.g., in Latin America) are available in the literature. Consulting or referencing these experiences could provide valuable insights for this study.”

Thank you for suggesting this helpful paper.
